# Multimodal Modeling of Neural Network Activity: Computing LFP, ECoG, EEG, and MEG Signals With LFPy 2.0

**DOI:** 10.3389/fninf.2018.00092

**Published:** 2018-12-18

**Authors:** Espen Hagen, Solveig Næss, Torbjørn V. Ness, Gaute T. Einevoll

**Affiliations:** ^1^Department of Physics, University of Oslo, Oslo, Norway; ^2^Faculty of Science and Technology, Norwegian University of Life Sciences, Ås, Norway; ^3^Department of Informatics, University of Oslo, Oslo, Norway

**Keywords:** modeling, neuron, neuronal network, local field potential, LFP, ECoG, EEG, MEG

## Abstract

Recordings of extracellular electrical, and later also magnetic, brain signals have been the dominant technique for measuring brain activity for decades. The interpretation of such signals is however nontrivial, as the measured signals result from both local and distant neuronal activity. In volume-conductor theory the extracellular potentials can be calculated from a distance-weighted sum of contributions from transmembrane currents of neurons. Given the same transmembrane currents, the contributions to the magnetic field recorded both inside and outside the brain can also be computed. This allows for the development of computational tools implementing forward models grounded in the biophysics underlying electrical and magnetic measurement modalities. LFPy (LFPy.readthedocs.io) incorporated a well-established scheme for predicting extracellular potentials of individual neurons with arbitrary levels of biological detail. It relies on NEURON (neuron.yale.edu) to compute transmembrane currents of multicompartment neurons which is then used in combination with an electrostatic forward model. Its functionality is now extended to allow for modeling of networks of multicompartment neurons with concurrent calculations of extracellular potentials and current dipole moments. The current dipole moments are then, in combination with suitable volume-conductor head models, used to compute non-invasive measures of neuronal activity, like scalp potentials (electroencephalographic recordings; EEG) and magnetic fields outside the head (magnetoencephalographic recordings; MEG). One such built-in head model is the four-sphere head model incorporating the different electric conductivities of brain, cerebrospinal fluid, skull and scalp. We demonstrate the new functionality of the software by constructing a network of biophysically detailed multicompartment neuron models from the Neocortical Microcircuit Collaboration (NMC) Portal (bbp.epfl.ch/nmc-portal) with corresponding statistics of connections and synapses, and compute *in vivo*-like extracellular potentials (local field potentials, LFP; electrocorticographical signals, ECoG) and corresponding current dipole moments. From the current dipole moments we estimate corresponding EEG and MEG signals using the four-sphere head model. We also show strong scaling performance of LFPy with different numbers of message-passing interface (MPI) processes, and for different network sizes with different density of connections. The open-source software LFPy is equally suitable for execution on laptops and in parallel on high-performance computing (HPC) facilities and is publicly available on GitHub.com.

## 1. Introduction

Ever since the 1950s, electrical recordings with sharp electrodes have been the most important method for studying *in vivo* activity in neurons and neural networks (Li and Jasper, 1953). In the last couple of decades, however, a host of new measurement methods has been developed and refined. One key development is the new generation of multicontact electrodes allowing for high-density electrical recordings across cortical laminae and areas, and the accompanying resurgence of interest in the low-frequency part of the extracellular signal, the “local field potential” (LFP) (Buzsáki, 2004; Buzsáki et al., 2012; Einevoll et al., 2013). The LFP is a population measure reflecting how dendrites integrate synaptic inputs, insight that cannot be obtained from measurement of spikes from a handful of neurons (Einevoll et al., 2013). Many new optical techniques for probing cortical activity have also been developed. Of particular interest is *two-photon calcium imaging*, which can measure the action potentials of individual neurons deep into cortical tissue (Helmchen and Denk, 2005), and *voltage-sensitive dye imaging (VSDI)*, which measures the average membrane potential across dendrites close to the cortical surface (Grinvald and Hildesheim, 2004). These add to the more established systems-level methods such as *electroencephalography* (*EEG*, Nunez and Srinivasan, 2006), which measures electrical potentials at the scalp, and *magnetoencephalography* (*MEG*, Hämäläinen et al., 1993) which measures the magnetic field outside the head.

A standard way of analyzing such neurophysiological data has been to look for correlations between measurements and how the subject is stimulated or behaves. For example, most of what we have learned about neural representation of visual information in visual cortex has come from receptive-field studies where the correlation between measured spikes and presented visual stimuli is mapped out (Hubel and Wiesel, 1959). The same approach has been used to map out the receptive fields for other sensory modalities (sound, touch, etc.), objects and celebrities (Quiroga et al., 2005), or the spatial location of the animal (O'Keefe and Dostrovsky, 1971; Hafting et al., 2005).

This purely statistical approach has limitations, however. For one, it only provides estimates for the neural representation and gives no direct insight into the circuit mechanisms giving rise to these representations. Secondly, the receptive field is inherently a *linear* measure of activity (Dayan and Abbott, 2001) and cannot in general capture non-linear network dynamics. The receptive field in primary visual cortex depends, for example, strongly on stimulation of the surrounding regions of visual space, an inherently non-linear effect (Blakemore and Tobin, 1972). For other cortical measurements, such as the LFP or VSDI, a statistical analysis is further complicated by the fact that the signals reflect activity in neuron populations rather than individual neurons (Petersen et al., 2003; Einevoll et al., 2013). This makes commonly-used statistical signal measures such as power spectra, correlation, coherence, and functional connectivity difficult to interpret in terms of activity in neurons and networks (Einevoll et al., 2013).

An alternative approach to a purely statistical analysis is, following in the tradition of physics, to formulate candidate hypotheses precisely in mathematics and then compute what each hypothesis would predict for the different types of measurements. Until now candidate cortical network models have typically only predicted spiking activity, thus preventing a proper comparison with measurements other than single-unit and multiunit recordings. To take full advantage of all available experiments, there is a need for biophysics-based forward-modeling tools for predicting other measurement modalities from candidate network models (Brette and Destexhe, 2012), that is, develop software that faithfully models the various types of measurements themselves. To facilitate the forward-modeling of extracellular potentials, both LFPs and spikes [i.e., either single-unit or multi-unit activity (MUA)], we developed LFPy (LFPy.readthedocs.io, Lindén et al., 2014), a Python tool using the NEURON simulator (Carnevale and Hines, 2006) and its Python interface (Hines et al., 2009).

The first release of LFPy (Lindén et al., 2014) implemented a well-established forward-modeling scheme where the extracellular potential is computed in a two-step process (Holt and Koch, 1999): First, the transmembrane currents of multicompartment neuron models are computed using NEURON. Second, the extracellular potential is computed as a weighted sum over contributions from the transmembrane currents from each compartment with weights prescribed by volume-conductor theory for an infinite volume conductor. In LFPy these functions are provided by a set of Python classes that can be instantiated to represent the cell, synapses, stimulation devices and extracellular electric measurement devices. By now this forward-model method has been used in a number of studies, for example to model extracellular spike waveforms (Holt and Koch, 1999; Gold et al., 2006, 2007; Pettersen and Einevoll, 2008; Pettersen et al., 2008; Franke et al., 2010; Schomburg et al., 2012; Thorbergsson et al., 2012; Reimann et al., 2013; Hagen et al., 2015; Ness et al., 2015; Cserpán et al., 2017; Miceli et al., 2017), LFP signals (Pettersen et al., 2008; Lindén et al., 2010, 2011; Gratiy et al., 2011; Makarova et al., 2011; Schomburg et al., 2012; Łęski et al., 2013; Martín-Vázquez et al., 2013, 2015; Reimann et al., 2013; Głąbska et al., 2014, 2016; Mazzoni et al., 2015; Sinha and Narayanan, 2015; Taxidis et al., 2015; Tomsett et al., 2015; Hagen et al., 2016, 2017; Ness et al., 2016, 2018) and recently axonal LFP contributions (McColgan et al., 2017). Some of these used LFPy to predict extracellular potentials (Łęski et al., 2013; Lindén et al., 2014; Hagen et al., 2015, 2016, 2017; Mazzoni et al., 2015; Ness et al., 2015, 2016, 2018; Tomsett et al., 2015; Miceli et al., 2017; Luo et al., 2018), while in Heiberg et al. (2016) LFPy was used to construct a small-world LGN network without predictions of extracellular potentials. Further, in Uhlirova et al. (2016) LFPy was used to compute neuronal membrane potentials.

**Figure 1:**
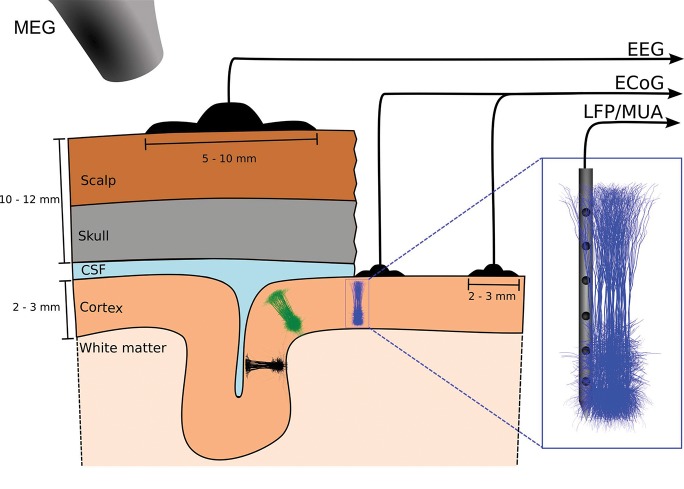
Illustration of measurement signals computed by LFPy 2.0. The figure illustrates the EEG, ECoG, LFP/MUA (linear multielectrode) and MEG recordings of electrical and magnetic signals stemming from populations of cortical neurons. Here three separate cortical populations are depicted. EEG electrodes are placed on the scalp, ECoG electrodes on the cortical surface, while the LFP and MUA both are recorded by electrodes placed inside cortex. In MEG the tiny magnetic fields stemming from brain activity is measured by SQUIDs placed outside the head. The MUA signal, that is, the high-frequency part of the recorded extracellular potential inside cortex, measures spikes from neurons in the immediate vicinity of the electrode contact, typically less than 100 μm away (Buzsáki, 2004; Pettersen and Einevoll, 2008; Pettersen et al., 2008). The “mesoscopic” LFP and ECoG signals will typically contain information from neurons within a few hundred micrometers or millimeters from the recording contact (Einevoll et al., 2013), while the “macroscopic” EEG and MEG signals will have contributions from cortical populations even further away (Hämäläinen et al., 1993; Nunez and Srinivasan, 2006).

**Figure 2:**
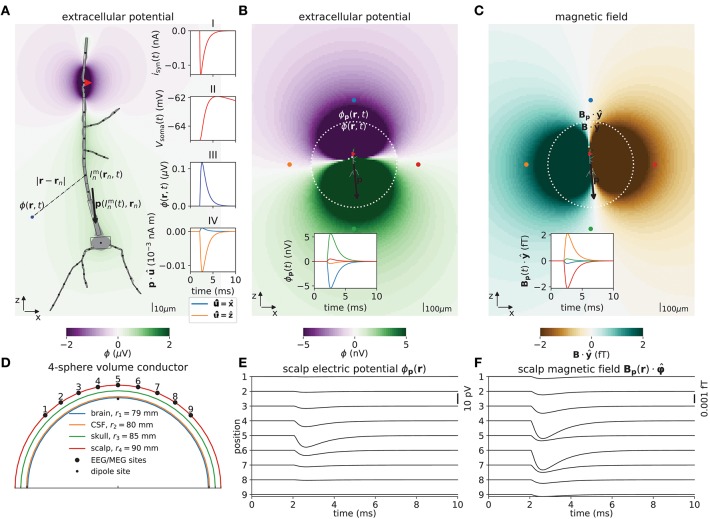
Illustrations of forward model, dipole approximation, EEG and MEG model. **(A)** Illustration of forward-modeling scheme for extracellular potentials from multicompartment neuron models. The gray shape illustrates soma and dendrites of a 3D-reconstructed neuron morphology and the equivalent multicompartment model. A single synaptic input current *i*_syn_(*t*) (red triangle, inset axes I) results in a deflection of the membrane voltage throughout the morphology, including at the soma (*V*_soma_(*t*), inset axes II). LFPy allows for computing extracellular potentials ϕ in arbitrarily chosen extracellular locations **r** (inset axes III) from transmembrane currents ($I_{n}^{\text{m}}\left({\text{r}}_{n},t\right)$), as well as the components of the current dipole moment **p** (black arrow, inset axes IV). Compartments are indexed *n*, **r**_*n*_ denote compartment positions. The image plot shows the extracellular potential in the *xz*-plane at the time of the largest synapse current magnitude (*t* = 2.25 ms). **(B)** Illustration of the extracellular electric potential calculated both from the current dipole moment and transmembrane currents for the situation in **(A)**. Within a radius *r* < 500 μm from the “center of areas” (see below) of the morphology the panel shows extracellular potentials ϕ(**r**) predicted using the line-source method, while outside this radius the panel shows extracellular potentials ϕ_**p**_(**r**) predicted from the current dipole moment (**p**, black arrow). Here, an assumption of an homogeneous (same everywhere) and isotropic (same in all directions) extracellular conductivity was used. The ‘center of areas‘ was defined as $\overset{n^{\text{seg}}}{\underset{n=1}{\sum }}A_{n}{\text{r}}_{n}/\overset{n^{\text{seg}}}{\underset{n=1}{\sum }}A_{n}$ where *A*_*n*_ denotes compartment surface area. The time *t* = 2.25 ms as in **(A)**. The inset axis shows the potential as function of time in the four corresponding locations (at |**R**| = 750 μm) surrounding the morphology (colored circular markers). **(C)** Visualization of magnetic field component ${\text{B}}_{\text{p}}\cdot \hat{\text{y}}$ (*y*-component) computed from the current dipole moment, outside a circle of radius *r* = 500 μm (as in **B**). Inside the circle, we computed the same magnetic field component from axial currents. The inset axis shows the *y*-component of the magnetic field as function of time in the four corresponding locations (at |**R**| = 750 μm) surrounding the morphology (circular markers). **(D)** Illustration of upper half of the four-sphere head model used for predictions of EEG scalp potentials from electric current dipole moments. Each spherical shell with outer radii *r* ∈ {*r*_1_, *r*_2_, *r*_3_, *r*_4_} has piecewise homogeneous and isotropic conductivity σ_e_ ∈ {σ_1_, σ_2_, σ_3_, σ_4_}. The EEG/MEG sites numbered 1–9 mark the locations where electric potentials and magnetic fields are computed, each offset by an arc length of *r*_4_π/16 in the *xz*-plane. The current dipole position was θ = φ = 0, *r* = 78 mm (in spherical coordinates). **(E)** Electric potentials on the outer scalp-layer positions 1-9 in **(D)**. **(F)** Tangential component of the magnetic field ${\text{B}}_{\text{p}}\cdot \hat{\varphi }$ in positions 1–9. (Note that at position 5, the unit vector $\hat{\varphi }$ is defined to be directed in the positive *y*-direction).

Here we present a substantially extended version of LFPy, termed LFPy 2.0, including several new features, that is, support for (i) simulations of networks of multicompartmental neuron models, (ii) computation of LFP/MUA with anisotropic electrical conductivity, (iii) computation of LFP/MUA in the presence of step-wise varying electrical conductivity (such as at the interface between cortical gray matter and white matter), (iv) computation of ECoG signals (i.e., electrical potentials recorded at the cortical surface), (v) computation of EEG signals, and (vi) computation of MEG signals, see illustration in Figure 1. To illustrate the computation of these measures by LFPy 2.0 we show in Figure 2 the LFP, EEG, and MEG signals generated by a single synaptic input onto a single simplified “pyramidal” neuron. As both electric and magnetic signals sum linearly, the recorded signals in real applications will stem from the sum of a large number of such contributions.

Potential uses of LFPy 2.0 include (but are not limited to): Comparison of candidate neuron and network models with arbitrary levels of detail to experiments in order to aid the interpretation of experimental data, validation of data analysis methods by testing them on synthetic (model-based) measurements with known underlying ground truth, and comparison of model predictions from different types of models with different levels of detail.

The manuscript is organized as follows: In section 2 we first review the biophysical forward-modeling scheme used to predict extracellular potentials in different volume-conductor models. Then we describe calculations of current dipole moments and corresponding calculation of EEG and MEG signals. We further describe the implementation of an example network using available data and biophysically detailed cell models from the Blue Brain Project's Neocortical Microcircuit Collaboration (NMC) Portal, and various technical details. In section 3 we investigate the outcome of our example parallel network simulation and corresponding measurements, and assess parallel performance of LFPy when running on HPC facilities. In section 4 we outline implications of this work and discuss possible future applications and developments of the software. In the Appendix we describe new LFPy classes and corresponding code examples for set-up of networks.

## 2. Methods

### 2.1. Multicompartment Modeling

#### 2.1.1. Calculation of Transmembrane Currents

The origin of extracellular potentials is mainly transmembrane currents (Buzsáki et al., 2012; Einevoll et al., 2013), even though diffusion of ions in the extracellular space alone also can give rise to such potentials (Halnes et al., 2016). In the presently (and frequently) used forward modeling approach, these transmembrane currents are obtained from spatially discretized multicompartment neuron models (De Schutter and Van Geit, 2009) which allow for high levels of biophysical and morphological detail. Such models have historically been used to model spatiotemporal variations in the membrane voltages *V*^m^(*x, t*), where *x* denotes the position along an unbranched piece of dendritic cable. From this cable theory it also follows that the transmembrane current density, that is, the transmembrane current per unit length of membrane, for any smooth and homogeneous cable section is given by (Koch, 1999):

(1) $$\begin{array}{l} i^{\text{m}}\left(x,t\right)=\frac{1}{r^{\text{i}}}\frac{\partial^{2}V^{\text{m}}\left(x,t\right)}{\partial x^{2}}\;, \end{array}$$

where *r*^i^ represents the axial resistance per unit length along the cable. Assuming a homogeneous current density per unit length *i*^m^ along a single compartment with length Δ*s*, the total transmembrane current *I*^m^ = *i*^m^Δ*s*.

As in the first release of LFPy (Lindén et al., 2014), we rely on the NEURON simulation environment (Carnevale and Hines, 2006) to compute transmembrane currents. As of NEURON v7.4, a faster and direct method of accessing transmembrane currents is provided through its CVode.use_fast_imem() method, which we now utilize in an exclusive manner. NEURON's “extracellular” mechanism is thus no longer used to predict extracellular potentials (cf. Lindén et al., 2014, section 5.6). Note, however, that this mechanism itself is still used when an external extracellular potential is imposed as a boundary condition outside each compartment using the Cell.insert_v_ext() class method.

#### 2.1.2. Calculation of Axial Currents

To compute the magnetic fields stemming from electrical activity in neurons, the axial currents within cells are needed (Hämäläinen et al., 1993). The axial current for the cable is given by (Koch, 1999):

(2) $$\begin{array}{l} I^{\text{a}}\left(x,t\right)=-\frac{1}{r^{\text{i}}}\frac{\partial V^{\text{m}}\left(x,t\right)}{\partial x}\;. \end{array}$$

Assuming homogeneous axial current density between the midpoints of two neighboring compartments *n* and *n* + 1 along the cable, one may obtain the axial current from Ohm's law:

(3) $$\begin{array}{l} I_{n,n+1}^{\text{a}}\left(t\right)=\frac{V_{n+1}^{\text{m}}\left(t\right)-V_{n}^{\text{m}}\left(t\right)}{r_{n,n+1}^{\text{i}}\Delta s_{n,n+1}}=\frac{V_{n+1}^{\text{m}}\left(t\right)-V_{n}^{\text{m}}\left(t\right)}{R_{n,n+1}^{\text{i}}}\;. \end{array}$$

Here, $V_{n}^{\text{m}}$ and $V_{n+1}^{\text{m}}$ are the compartment midpoint membrane potentials, $r_{n,n+1}^{\text{i}}$ the axial resistance per unit length between the two compartments, Δ*s*_*n,n*+1_ the distance between compartment midpoints and $R_{n,n+1}^{\text{i}}$ the corresponding axial resistance.

Further, we outline how axial currents from complex reconstructed neuron morphologies are calculated in LFPy 2.0, and provide the technical implementation details in Algorithm A1 in the Appendix. For a more comprehensive explanation, see Næss (2015). The corresponding implementation is in LFPy 2.0 provided by the class method Cell.get_axial_currents_from_vmem().

**Figure 3:**
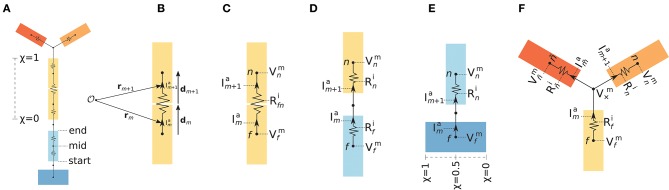
Axial currents in multicompartment neuron models. **(A)** Schematic illustration of sections (colored rectangles), segments and equivalent electric circuit of a simplified multicompartment neuron model. The relative length χ varies between 0 and 1 from start- to end-point of each section. **(B)** Axial current line element vectors (**d**_*m*_, **d**_*m*+1_) and corresponding midpoints (**r**_*m*_, **r**_*m*+1_) of axial currents ($I_{m}^{\text{a}},I_{m+1}^{\text{a}}$) between two connected segments. **(C)** Axial currents ($I_{m}^{\text{a}},I_{m+1}^{\text{a}}$), membrane potentials ($V_{f}^{\text{m}},V_{n}^{\text{m}}$), and axial resistance ($R_{fn}^{\text{i}}$) in equivalent electric circuit for a parent segment *f* and child segment *n* in a single section. **(D)** Similar to panel B, but parent and child segments belong to two different sections. The total series resistance is here $R_{f}^{\text{i}}+R_{n}^{\text{i}}$. **(E)** Illustration of the case where the child segment *n* is connected to a point χ = 0.5 on the parent section. For children connected at χ ∈ 〈0, 1〉 the voltage difference ($V_{n}^{\text{m}}-V_{f}^{\text{m}}$) is only across the child segment axial resistance $R_{n}^{\text{i}}$, but the (virtual) current from the node connecting the child start point to the parent midpoint $I_{m}^{\text{a}}$ is still accounted for. **(F)** Illustration of axial currents at branch point between different sections of the morphology. The child segment *n* has one parent *f* and one sibling indexed by ñ, where $V_{\times }^{\text{m}}$ denotes the virtual membrane potential at the node connecting the parent end-point to the children start-points. $V_{ñ}^{\text{m}}$ is the voltage in the midpoint of the sibling segment, while $R_{ñ}^{\text{i}}$ and $I_{\tilde{m}}^{\text{a}}$ denotes the axial resistance and current between the sibling midpoint and the branch point.

In NEURON, a *section* is a continuous piece of cable split into an arbitrary number of *segments* (compartments) indexed by *n*. Morphologies with branch points must therefore be represented by more than one section. We here denote the relative length from start to end point of each section by χ ∈ [0, 1], see Figure 3A. All segments within the morphology except the initial segment of the *root* section (typically the somatic section) have a *parent* segment indexed by *f*. Each segment in a section can have an arbitrary number of *child* segments, thus a parent segment is the segment which connects to the start point of a *child* segment. We also distinguish between start-, mid- and end-point coordinates of each segment (Figure 3A).

In Figures 3B,C we illustrate the simplest possible calculation of axial current between the midpoints of two neighboring segments *f* and *n* belonging to the same section. Their corresponding membrane voltages are $V_{f}^{\text{m}}$ and $V_{n}^{\text{m}}$, separated by a total (series) axial resistance $R_{fn}^{\text{i}}$. From NEURON we can easily obtain the axial resistance between the segment midpoint and the segment's parent node. The parent node is here the midpoint of the parent segment, as the child and parent belong to the same section. Therefore, NEURON gives us the total axial resistance $R_{fn}^{\text{i}}$ directly, in this case. The axial current magnitude between segment midpoints is then trivial to compute using Ohm's law (Equation 3), but as the currents flowing within segments *f* and *n* may not lie on the same axis, we differentiate between the current magnitudes $I_{m}^{\text{a}}$ and $I_{m+1}^{\text{a}}$, their axial line element vectors **d**_*m*_ and **d**_*m*+1_, and the midpoints of each **r**_*m*_ and **r**_*m*+1_ (Figure 3C). The corresponding current indices are denoted by *m* and *m*+1 as detailed in Algorithm A1 (Appendix).

Figure 3D represents the case where the parent and child segments *f* and *n* belong to different sections. The child segment is here the *bottom segment* in a section, and it is connected to the end point of *f*. As the parent node (the node the child segment connects to on the parent segment) is here located between the two segments, NEURON does in this case not give us the total axial resistance directly. Instead, the total (series) axial resistance $R_{fn}^{\text{i}}=R_{f}^{\text{i}}+R_{n}^{\text{i}}$ must first be computed to estimate the axial current. $R_{f}^{\text{i}}$ is here the resistance between the parent midpoint and the connecting node, and $R_{n}^{\text{i}}$ the resistance between the parent node and the segment midpoint.

NEURON allows child sections to be connected anywhere along the parent section (χ ∈ [0, 1]). Illustrated in Figure 3E, a child segment is connected to the point χ = 0.5 and the axial resistance in the parent segment does not enter the calculation of axial current magnitude. LFPy 2.0 still accounts for a virtual axial current $I_{m}^{\text{a}}$ from the parent mid point to the child start point. These virtual currents ensure that the total current dipole moments computed either from transmembrane currents or from axial currents are identical (see section 2.3.1 for details).

At morphology branch points, several child segments may protrude from a parent segment as illustrated in Figure 3F. As the segment *n* and its sibling *ñ* both share the same parent *f*, we estimate the potential $V_{\times }^{\text{m}}$ at the branch node using Ohm's law and Kirchhoff's current law, accounting for the axial resistivities $\left(R_{f}^{\text{i}},\;R_{n}^{\text{i}},\;R_{\tilde{n}}^{\text{i}}\right)$ and potentials $\left(V_{f}^{\text{m}},\;V_{n}^{\text{m}},\;V_{\tilde{n}}^{\text{m}}\right)$, in order to compute the corresponding axial currents $I_{m}^{\text{a}}$ and $I_{m+1}^{\text{a}}$. The full procedure presently used for computing axial currents in LFPy 2.0 for the cases illustrated in Figures 3B–F is provided in full detail in Algorithm A1 (Appendix).

### 2.2. Forward Modeling of LFP and MUA Signals

The relation between transmembrane currents and extracellular potentials is calculated based on volume conduction theory (Nunez and Srinivasan, 2006; Einevoll et al., 2013). At the relatively low frequencies relevant in neurophysiology (below a few thousand hertz), this derivation is simplified by omitting terms with time derivatives in Maxwell's equations (quasistatic approximation, Hämäläinen et al., 1993, p. 426). Further, the extracellular medium is in all situations considered below assumed to be ohmic, that is, linear and frequency-independent (Pettersen et al., 2012; Einevoll et al., 2013; Miceli et al., 2017).

#### 2.2.1. Homogeneous and Isotropic Media

We first consider the simplest situation, where the medium is *homogeneous*, that is, the same in all positions corresponding to an infinite volume conductor, and *isotropic*, that is, the same electrical conductivity in all directions. The medium is then represented by a scalar extracellular conductivity σ_e_. The extracellular potential ϕ(**r**, *t*) at position **r** and time *t* is then given by (Nunez and Srinivasan, 2006; Lindén et al., 2014)

(4) $$\phi (r,t)=\frac{1}{4\pi \sigma_{\text{e}}}\frac{I(t)}{\left|r-r^{\prime }\right|}\;,$$

where *I*(*t*) represents a time-varying point current source at position **r′**. For transmembrane currents $I_{jn}^{\text{m}}\left(t\right)$ of individual compartments $n\in \left[1,n_{j}^{\text{seg}}\right]$ of all cells *j* in a population of *N* cells, the extracellular potential can be computed as the linear sum of their contributions as

(5) $$\begin{array}{l} \phi \left(\text{r},t\right)=\frac{1}{4\pi \sigma_{\text{e}}}\overset{N}{\underset{j=1}{\sum }}\overset{n_{j}^{\text{seg}}}{\underset{n=1}{\sum }}\frac{I_{jn}^{\text{m}}\left(t\right)}{\left|\text{r}-{\text{r}}_{jn}\right|}\;, \end{array}$$

but only under the assumption that each transmembrane current can be represented as a discrete point in space. This point-source assumption can be used in LFPy by supplying the keyword argument and value method=“pointsource” to the RecExtElectrode class (Lindén et al., 2014).

As a homogeneous current distribution along each cylindrical compartment is assumed, we may employ the *line-source* approximation for somatic and dendritic compartments (Holt and Koch, 1999). The formula is obtained by integrating 4 along the center axis of each cylindrical compartment *n*, and by summing over contributions from every $n_{j}^{\text{seg}}$ compartment of all *N* cells (Holt and Koch, 1999; Pettersen and Einevoll, 2008; Lindén et al., 2014):

(6) $$\begin{array}{l} \phi \left(r,t\right)=\frac{1}{4\pi \sigma_{\text{e}}}\sum_{j=1}^{N}\sum_{n=1}^{n_{j}^{\text{seg}}}I_{jn}^{\text{m}}\left(t\right)\int \frac{1}{\left|r-r_{jn}\right|}dr_{jn}\\ \;=\frac{1}{4\pi \sigma_{\text{e}}}\sum_{j=1}^{N}\sum_{n=1}^{n_{j}^{\text{seg}}}\frac{I_{jn}^{\text{m}}\left(t\right)}{\Delta s_{jn}}\ln\left|\frac{\sqrt{h_{jn}^{2}+r_{⊥jn}^{2}}-h_{jn}}{\sqrt{l_{jn}^{2}+r_{⊥jn}^{2}}-l_{jn}}\right|\;. \end{array}$$

Compartment length is denoted Δ*s*_*jn*_, perpendicular distance from the electrode point contact to the axis of the line compartment is denoted *r*_⊥*jn*_, longitudinal distance measured from the start of the compartment is denoted *h*_*jn*_, and longitudinal distance from the other end of the compartment is denoted *l*_*jn*_ = Δ*s*_*jn*_ + *h*_*jn*_. The corresponding keyword argument and value to class RecExtElectrode is method=“linesource” (Lindén et al., 2014).

A final option in LFPy is however to approximate the typically more rounded soma compartments as spherical current sources, thus the line-source formula (Equation 6) for dendrite compartments is combined with the point-source equation (Equation 4), obtaining (Lindén et al., 2014):

(7) $$\begin{array}{l} \phi \left(r,t\right)=\frac{1}{4\pi \sigma_{\text{e}}}\sum_{j=1}^{N}\left(\frac{I_{j,\text{soma}}^{\text{m}}(t)}{\left|r-r_{j,\text{soma}}\right|}+\sum_{n=2}^{n_{j}^{\text{seg}}}\int \frac{I_{jn}^{\text{m}}(t)}{\left|r-r_{jn}\right|}dr_{jn}\right)\\ \;=\frac{1}{4\pi \sigma_{\text{e}}}\sum_{j=1}^{N}\left(\frac{I_{j,\text{soma}}^{\text{m}}(t)}{\left|r-r_{j,\text{soma}}\right|}+\sum_{n=2}^{n_{j}^{\text{seg}}}\frac{I_{jn}^{\text{m}}(t)}{\Delta s_{jn}}\ln\left|\frac{\sqrt{h_{jn}^{2}+r_{⊥jn}^{2}}-h_{jn}}{\sqrt{l_{jn}^{2}+r_{⊥jn}^{2}}-l_{jn}}\right|\right)\;. \end{array}$$

The corresponding keyword argument and value is method=“soma_as_point”.

If the distance between current sources and electrode contacts is smaller than the radius of the segment, unphysical singularities may occur in the computed extracellular potential. Singularities are in LFPy automatically prevented by either setting *r*_⊥*jn*_ or |**r** − **r**_*jn*_| equal to the cylindrical compartment radius dependent on the choice of line or point sources.

Electrode contacts of real recording devices have finite spatial extents. A good approximation to the electric potential across the uninsulated surface of metal electrode contact is obtained by computing the spatially averaged electric potential (Robinson, 1968; Nelson et al., 2008; Nelson and Pouget, 2010; Ness et al., 2015), in particular for current sources being located at distances larger than approximately one electrode radius (Ness et al., 2015). The *disc-electrode* approximation to the potential (Camuñas-Mesa and Quiroga, 2013; Lindén et al., 2014; Ness et al., 2015)

(8) $$\begin{array}{l} \phi_{\text{disc}}\left(\text{u},t\right)=\frac{1}{A_{S}}\iint_{S}\phi \left(\text{u},t\right)d^{2}r\approx \frac{1}{m}\overset{m}{\underset{h=1}{\sum }}\phi \left({\text{u}}_{h},t\right)\;, \end{array}$$

is incorporated in LFPy, with corresponding parameters for contact radius *r*_contact_, number *m* of random points **u**_*h*_ on the flat, circular electrode contact surface when averaging (Lindén et al., 2014). The surface normal vector for each electrode contact must also be specified.

#### 2.2.2. Discontinuous and Isotropic Media

Above we described the case for an infinite volume conductor, that is, a constant extracellular conductivity σ_e_, as implemented in the initial LFPy release (Lindén et al., 2014). For cases where σ_e_ vary with position, i.e., σ_e_ = σ_e_(**r**), such as for cortical *in vivo* recordings close to the cortical surface (Einevoll et al., 2007) or *in vitro* recordings using microelectrode arrays (MEAs) (Ness et al., 2015), this approximation does not generally hold. Instead a generalized Poisson equation must be solved (Nicholson and Freeman, 1975):

(9) $$\begin{array}{l} \nabla \cdot \left(\sigma_{\text{e}}\left(\text{r}\right)\nabla \phi \left(\text{r},t\right)\right)=-C\left(\text{r},t\right), \end{array}$$

where *C*(**r**, *t*) is the current-source density. This equation can always be solved numerically by means of the Finite Element Method (FEM) (McIntyre and Grill, 2001; Ness et al., 2015) or other mesh-based methods (see for example Tveito et al., 2017).

In the special case where the conductivity σ_e_ is discontinuous in a single direction, that is, a constant conductivity in the *xy*-plane and a piecewise constant σ_e_(*z*) in the *z*-direction, the ‘Method-of-Images’ (MoI) can be used to make analytical formulas for the extracellular potentials, analogous to 4–7 above (Nicholson and Llinas, 1971; Nunez and Srinivasan, 2006; Ness et al., 2015). When applicable, these formulas substantially simplify the modeling of the extracellular potentials compared to FEM modeling.

*Electrical potentials across microelectrode arrays (MEAs):* The first MoI application is to model recordings in a MEA setting where a slice of brain tissue is put on an insulating recording chip (MEA-chip) and covered with saline (Hagen et al., 2015; Ness et al., 2015). In this three-layer situation separate conductivity values are assigned to the topmost saline layer conductivity σ_S_ for *z* ∈ [*h*, ∞], the middle tissue layer conductivity σ_T_ for *z* ∈ [0, *h*) and the lowermost electrode σ_G_ for *z* ∈ [−∞, 0). The parameter *h* denotes the thickness of the middle tissue layer. The corresponding implementation is provided by the class RecMEAElectrode, and has at present the limitations that all current sources (segments) must be contained on the interval *z* ∈ [0, *h*), and that the line-source approximation can only be used when σ_G_ = 0 and when computing extracellular potentials for *z* = 0. For other forward-model configurations (for example for 0 ≤ *z* ≤ *h* and/or σ_G_ > 0) the point-source approximation can be used. For a detailed derivation of the MoI with two planar electrical boundaries, see Equation (4) in Ness et al. (2015). A corresponding example is provided with LFPy 2.0 (example_MEA.py) which illustrates the computation of extracellular potentials as recorded by a MEA following synaptic activation of a pyramidal cell model.

*Electrical potentials close to cortical surface:* The second MoI application is to model *in vivo* recordings of electrical potentials at or immediately below the cortical surface, that is, the interface between cortical gray matter and dura. Here the extracellular conductivity above the cortical surface σ_S_ can be higher or lower than the conductivity in cortical gray matter σ_T_ depending on how the measurements are done, for example whether saline or oil is used to cover an inserted laminar electrode (Einevoll et al., 2007). Such a conductivity jump will affect both the electrical potential recorded at the cortical surface (ECoG recording) as well as the potentials recorded in the top cortical layers (Pettersen et al., 2006). This can be modeled with the same framework as above, that is, by using the class RecMEAElectrode, with the cortical surface at height *h*, while ignoring the lower planar boundary by setting σ_G_ = σ_T_. In this situation the potential at or below the cortical surface at position (*x, y, z*) for a current source, *I*(*t*), positioned at (*x*′, *y*′, *z*′) is given by (Nunez and Srinivasan, 2006; Pettersen et al., 2006; Ness et al., 2015) as:

(10) $$\begin{array}{l} \phi (x,y,z,t)=\frac{I(t)}{4\pi \sigma_{\text{T}}}(\frac{1}{\sqrt{{\left(x-x^{\prime }\right)}^{2}+{\left(y-y^{\prime }\right)}^{2}+{\left(z-z^{\prime }\right)}^{2}}}\\ \;+\frac{\sigma_{\text{T}}-\sigma_{\text{S}}}{\sigma_{\text{T}}+\sigma_{\text{S}}}\frac{1}{\sqrt{{\left(x-x^{\prime }\right)}^{2}+{\left(y-y^{\prime }\right)}^{2}+{\left(z+z^{\prime }-2h\right)}^{2}}}). \end{array}$$

This approach assumes a flat cortical surface. Note, however, that in LFPy 2.0 the ECoG signal can also be modeled by means of the four-sphere EEG head model as described below in section 2.3.4. An example is provided with LFPy 2.0 (example_ECoG.py) which illustrates extracellular potentials recorded in the cortex and at the cortical surface following activation of multiple synapses distributed across a pyramidal cell model.

Electrical potentials in spherical conductor:

LFPy 2.0 also incorporates a spherical conductor model, adapted from Deng (2008), where the conductivity is constant within the sphere and constant outside (class OneSphereVolumeConductor). Note that this model is applicable for monopolar current sources, unlike the more complex multi-sphere head models described below in section 2.3 which only apply to dipolar current sources. Although not pursued here, one application of this volume-conductor model could possibly be modeling of LFPs measured in spheroidal brain nuclei.

#### 2.2.3. Homogeneous and Anisotropic Media

For homogeneous media, that is, when the extracellular conductivity is the same at all positions, we also added support for anisotropic media (Nicholson and Freeman, 1975). In this case the extracellular conductivity in 9 must be replaced by a rank 2 (3 × 3) tensor where the diagonal elements are σ_*x*_, σ_*y*_, and σ_*z*_ and the off-diagonal elements are zero (Nicholson and Freeman, 1975). This could for example be used to mimic experimental observations of such anisotropy in cortex (Goto et al., 2010), that is, electric currents flow with less resistance along the depth direction (*z*-direction) than in the lateral directions (*x, y*-directions). In this case σ_*z*_ > σ_*x*_ = σ_*y*_ (Ness et al., 2015). The corresponding implementation is based on the description and implementation provided by Ness et al. (2015), and is in LFPy presently supported by the class RecExtElectrode, but not the class RecMEAElectrode.

### 2.3. Forward Modeling of EEG, ECoG, and MEG Signals From Current Dipoles

The forward modeling of EEG and MEG signals from current dipoles has a long history (Hämäläinen et al., 1993; Nunez and Srinivasan, 2006). Here the EEG contacts and the MEG magnetometers are located so far away from the neural sources that only the current dipole moments contribute to the measured signals, that is, the contributions from higher-order current multipoles are negligible. From charge conservation, it follows that current monopoles do not exist. To compute the contribution to EEG and MEG signals from detailed neuron models, we thus first need to compute single-neuron current dipole moments as described in section 2.3.1. Next these must be combined with appropriate volume-conductor models for the head.

In LFPy 2.0 we include two “head” models for computing EEG signals from current dipole moments: the (very simplified) infinite homogenous volume-conductor model (section 2.3.2), and the much more involved four-sphere head model where the brain tissue, cerebrospinal fluid (CSF), skull and scalp are represented with different values for the electrical conductivity (Nunez and Srinivasan, 2006; Næss et al., 2017), cf. section 2.3.3. For the MEG signals the forward model is simpler as the magnetic permeability is the same throughout the head as in free space (Hämäläinen et al., 1993). In LFPy 2.0 we include simulation code for computing neural contributions to MEG signals applicable for all head models with spherically-symmetric electrical conductivities, for example, the four-sphere head model, cf. section 2.3.5. While these head models allow for direct calculation of EEG and MEG signals from neurons, it should be noted that the computed current dipole moments also can be used for subsequent calculation of EEG and MEG signals by means of boundary element (BEM) or finite element models (FEM) with anatomically detailed head models (He et al., 2002; Bangera et al., 2010; DeMunck et al., 2012),(Huang et al., 2016).

#### 2.3.1. Calculation of Current Dipole Moments

*Current dipole moments from transmembrane currents:* The current dipole moment from a single neuron can be computed from transmembrane currents as (Lindén et al., 2010):

(11) $$\begin{array}{l} \text{p}\left(t\right)=\overset{n^{\text{seg}}}{\underset{n=1}{\sum }}{\text{r}}_{n}I_{n}^{\text{m}}\left(t\right)\;, \end{array}$$

where $I_{n}^{\text{m}}$ is the transmembrane current at time *t* from compartment *n* at position **r**_*n*_. For a population of *N* cells with $n_{j}^{\text{seg}}$ compartments each, the current dipole moment at discrete time steps can be formulated as the matrix product:

(12) $$\left[\begin{array}{ccc} p_{x}(0) & p_{y}(0) & p_{z}(0)\\ p_{x}(dt) & p_{y}(dt) & p_{z}(dt)\\ \vdots & \vdots & \vdots \\ p_{x}(T) & p_{y}(T) & p_{z}(T) \end{array}\right]={\left[\begin{array}{cccc} I_{11}^{\text{m}}(0) & I_{11}^{\text{m}}(dt) & \ldots & I_{11}^{\text{m}}(T)\\ I_{12}^{\text{m}}(0) & I_{12}^{\text{m}}(dt) & \ldots & I_{12}^{\text{m}}(T)\\ \vdots & \vdots & \ddots & \vdots \\ I_{jn}^{\text{m}}(0) & I_{jn}^{\text{m}}(dt) & \ldots & I_{jn}^{\text{m}}(T)\\ \vdots & \vdots & \ddots & \vdots \\ I_{Nn_{j}^{\text{seg}}}^{\text{m}}(0) & I_{Nn_{j}^{\text{seg}}}^{\text{m}}(dt) & \ldots & I_{Nn_{j}^{\text{seg}}}^{\text{m}}(T) \end{array}\right]}^{T}\left[\begin{array}{ccc} r_{11}^{\left(x\right)} & r_{11}^{\left(y\right)} & r_{11}^{\left(z\right)}\\ r_{12}^{\left(x\right)} & r_{12}^{\left(y\right)} & r_{12}^{\left(z\right)}\\ \vdots & \vdots & \vdots \\ r_{jn}^{\left(x\right)} & r_{jn}^{\left(y\right)} & r_{jn}^{\left(z\right)}\\ \vdots & \vdots & \vdots \\ r_{Nn_{j}^{\text{seg}}}^{\left(x\right)} & r_{Nn_{j}^{\text{seg}}}^{\left(y\right)} & r_{Nn_{j}^{\text{seg}}}^{\left(z\right)} \end{array}\right]\;,$$

where *p*_*u*_(*t*) is the *u*-component (*u* ∈ {*x, y, z*}) of the current dipole moment at time *t* (thus $\text{p}\left(t\right)\equiv p_{x}\left(t\right)\hat{\text{x}}+p_{y}\left(t\right)\hat{\text{y}}+p_{z}\left(t\right)\hat{\text{z}}$), $I_{jn}^{\text{m}}\left(t\right)$ the transmembrane currents of segment *n* of cell *j* at time *t* and $r_{jn}^{\left(u\right)}$ the corresponding *u*-coordinates of each segment's midpoint. $\hat{\text{x}},\hat{\text{y}}$ and $\hat{\text{z}}$ denote the cartesian unit vectors. For more compact notation we here show the transpose (denoted by the raised *T*) of the matrix containing transmembrane currents. Note that the same formula may be used to also compute current dipole moments **p**_*j*_ of individual cells *j* (or subsets thereof) by slicing the corresponding matrix elements.

*Current dipole moments from axial currents:* Alternatively, the current dipole moment can be computed from axial currents between neighboring segments (see section 2.1.2). As an example, we consider a two-compartmental dendritic stick model, where segment *one* will act as a current sink, and segment *two* as a current source. The transmembrane current entering segment two $I_{2}^{\text{m}}$ will be the same as the axial current *I*^a^ between the two segments, which is also equal to the current leaving compartment one $I_{1}^{\text{m}}$, such that $I_{1}^{\text{m}}=-I_{2}^{\text{m}}=I^{\text{a}}$. An axial line element vector **d** represents the path traveled by the axial current, which corresponds to the displacement **r**_1_ − **r**_2_ between the compartment midpoints. From equation 11 it thus follows that the current dipole moment is:

(13) $$\begin{array}{l} \text{p}=\overset{2}{\underset{n=1}{\sum }}{\text{r}}_{n}I_{n}^{\text{m}}=I^{\text{a}}\text{d}. \end{array}$$

Multiplying each axial current with the respective current path gives a set of current dipoles:

(14) $$\begin{array}{l} {\text{p}}_{m}\left(t\right)=I_{m}^{\text{a}}\left(t\right){\text{d}}_{m}. \end{array}$$

Calculating sets of current dipole moments from neural simulations can be useful, for example for ECoG predictions (see section 2.3.4) or magnetic fields in proximity of the neuron (see section 2.4).

#### 2.3.2. EEG Signal for Homogeneous Volume Conductor

From eletrostatic theory we have that the electric potential outside a spatial distribution of current sinks and sources can be described by a multipole expansion $\phi \left(r\right)=C_{\text{monopole}}/R+C_{\text{dipole}}/R^{2}+C_{\text{quadrupole}}/R^{3}+C_{\text{octupole}}/R^{4}+\ldots \;,$ where *R* is the relative distance from the multipole to measurement location (and the coefficients *C* depends on the spherical angles). Due to charge conservation, current monopoles do not exist (Nunez and Srinivasan, 2006). For sufficiently large values of *R* where $C_{\text{dipole}}/R^{2}\gg \overset{\infty }{\underset{q=3}{\sum }}C_{q-\text{pole}}/R^{q}$, the electric potential of a neuron can be approximated solely from its current dipole moment, as contributions from quadrupolar and higher-order terms become negligible. The electric potential from a current dipole in an ohmic, homogeneous and isotropic medium is given by (Nunez and Srinivasan, 2006)

(15) $$\begin{array}{l} \phi_{\text{p}}=\frac{\text{p }\cdot \text{ R}}{4\pi \sigma_{\text{e}}R^{3}}\;, \end{array}$$

where **p** is the current dipole moment as defined above, σ_e_ the conductivity of the extracellular medium, **R** = **r** − **r**′ the displacement vector between dipole location **r**′ and measurement location **r**, and *R* = |**R**|. Predictions of extracellular potentials from current dipole moments in homogeneous media are provided by the class InfiniteVolumeConductor.

#### 2.3.3. EEG Signal in Four-Sphere Head Model

The computation of EEG signals assuming a homogeneous volume conductor model is obviously a gross approximation as it neglects the large variation in the extracellular conductivity in the head. In order to compute more realistic EEG signals from underlying neuronal sources, we implemented in LFPy 2.0 the inhomogeneous four-sphere head model in class FourSphereVolumeConductor. This model is composed of four concentric shells representing brain tissue, cerebrospinal fluid (CSF), skull and scalp, where the conductivity can be set individually for each shell (Srinivasan et al., 1998; Nunez and Srinivasan, 2006). Note that corrections to the original model formulation was recently provided in Næss et al. (2017). LFPy 2.0 incorporates this corrected four-sphere head model.

#### 2.3.4. ECoG Signal From Four-Sphere Head Model

The four-sphere head model is not restricted to EEG predictions, but can also be applied for modeling electric potentials in other layers of the inhomogeneous head model, such as ECoG signals at the interface between the brain tissue and the CSF. In contrast to EEG electrodes, however, the ECoG electrodes are located only micrometers away from the apical dendrites. The electrode's proximity to the neuronal source makes the four-sphere model a less obvious candidate model, as the model is based on the current dipole approximation, giving good predictions only when the measurement point is more than some dipole lengths away from the source (Lindén et al., 2010). However, in the FourSphereVolumeConductor class method calc_potential_from_multi_dipoles(), this problem can be avoided by taking advantage of the fact that electric potentials sum linearly in ohmic media: Instead of computing a single current dipole moment for the whole neuron, we compute multiple current dipole moments, one for each axial current, as described in section 2.3.1. Since these current dipoles have small enough source separations for the current dipole approximation to be applicable, we can compute the ECoG signal contribution from each current dipole moment separately, using the four-sphere model. The ECoG signal is finally predicted by summing up each contribution. The corresponding LFPy 2.0 example file is /examples/example_ECoG_4sphere.py.

#### 2.3.5. MEG Signals in Spherically-Symmetric Head Models

For spherically-symmetric head models the MEG signal can be computed from the current dipole moments set up by intracellular axial currents (Hämäläinen et al., 1993, p. 428). To compute magnetic fields **B**_**p**_ from current dipole moments we incorporated the special form of the magnetostatic Biot-Savart law (where magnetic induction effects are neglected) (Nunez and Srinivasan, 2006, Appendix C) given as:

(16) $$\begin{array}{l} {\text{B}}_{\text{p}}=\frac{\mu_{0}}{4\pi }\frac{\text{p }\times \text{ R}}{R^{3}}\;. \end{array}$$

As above, **p** is the dipole source, **R** = **r** − **r**′ the displacement between dipole location **r**′ and measurement location **r**, and *R* = |**R**|. For a detailed derivation of this expression see Hämäläinen et al. (1993). The magnetic field **B** is related to the commonly used quantity **H** (often also termed magnetic field) through **B** = μ_0_**H** + **M** = μ**H** where **M** is the magnetization and μ the magnetic permeability of the material. However, in biological tissues the magnetization **M** is very small, and μ is very close to the magnetic constant (i.e., the magnetic permeability of vacuum) μ_0_ (Hämäläinen et al., 1993). Predictions of magnetic signals are in LFPy 2.0 incorporated in the class MEG, which provides the method calculate_H in order to compute the magnetic field from a current dipole moment time series. Its output must be multiplied by μ to obtain the magnetic field **B_p_**.

Throughout this paper, we show for the four-sphere head model magnetic field components decomposed into tangential and radial components at different positions on spherical surfaces. The tangential components were computed in the direction of the angular unit vectors $\hat{\theta }=\cos\theta \cos\varphi \hat{\text{x}}+\cos\theta \sin\varphi \hat{\text{y}}-\sin\theta \hat{\text{z}}$ and $\hat{\varphi }=-\sin\varphi \hat{\text{x}}+\cos\varphi \hat{\text{y}}$ as $\text{B}\cdot \hat{\theta }$ and $\text{B}\cdot \hat{\varphi }$, respectively. The radial component was computed as ${\text{B}}_{\text{p}}\cdot \hat{\text{r}}$ where $\hat{\text{r}}$ denotes the radial unit vector from the center of the sphere in the direction of the contact. Furthermore, we also show tangential and radial components of the surface magnetic field where the underlying dipoles were rotated by an angle θ = π/2 around the *x*-axis, denoted ${\text{B}}_{R_{x}\left(\pi /2\right)\text{p}}\cdot \hat{\theta }$, ${\text{B}}_{R_{x}\left(\pi /2\right)\text{p}}\cdot \hat{\varphi }$ and ${\text{B}}_{R_{x}\left(\pi /2\right)\text{p}}\cdot \hat{\text{r}}$, respectively. For this purpose we used the rotation matrix

(17) $$\begin{array}{l} R_{x}\left(\frac{\pi }{2}\right)=\left[\begin{array}{ccc} 1 & 0 & 0\\ 0 & 0 & -1\\ 0 & 1 & 0 \end{array}\right]\; \end{array}$$

multiplied with the current dipole moment **p** in cartesian coordinates.

Note that experimental MEG equipment using gradiometers measure changes in the magnetic field across space in units of T/m (Hämäläinen et al., 1993). We here display the time-varying magnitude of magnetic fields in units of T.

### 2.4. Magnetic Signals Close to Neurons

Most studies of magnetic fields generated by neural activity have been based on MEG recordings where the neuronal sources are so distant from the magnetic-field sensors that the far-field dipole approximation in 16 can be applied. However, probes are also being developed for measuring magnetic fields in direct vicinity of the neurons (Barbieri et al., 2016; Caruso et al., 2017). To compute the magnetic fields in the vicinity of neurons, LFPy 2.0 also implements the relevant Biot-Savart law for this situation (Blagoev et al., 2007):

(18) $$B(r)=\frac{\mu_{0}}{4\pi }\sum_{m=1}^{m_{\text{a}}}I_{m}^{\text{a}}\frac{d_{m}\times (r-r_{m})}{|r-r_{m}{|}^{3}}\;.$$

This formula provides the magnetic field for *m*_a_ axial currents $I_{m}^{\text{a}}$ where **d**_*m*_ are axial line element vectors, and **r**_*m*_ the midpoint positions of each axial current. The use of this formula assumes that contributions to the magnetic fields from extracellular volume currents are negligible (Hämäläinen et al., 1993, p. 427). Predictions of magnetic signals from axial currents (or equivalently sets of current dipoles) are in LFPy 2.0 facilitated by the corresponding class method MEG.calculate_H_from_iaxial(). We show (in Figure 2) the *y*-components of the magnetic fields in vicinity of a model neuron computed as $\text{B}\cdot \hat{\text{y}}$ and ${\text{B}}_{p}\cdot \hat{\text{y}}$ respectively.

### 2.5. Description of Biophysically Detailed Network in Example Use Case

#### 2.5.1. Neuron Models

Our example network model presented in section 3 comprised about 5500 biophysically detailed multicompartment neurons obtained from The Neocortical Microcircuit Collaboration (NMC) Portal (https://bbp.epfl.ch/nmc-portal, Ramaswamy et al., 2015). The NMC portal provides NEURON code for about 1,000 different single-cell models as well as connectivity data of a reconstruction and simulation of a rat somatosensory cortex column (Markram et al., 2015).

**Figure 4:**
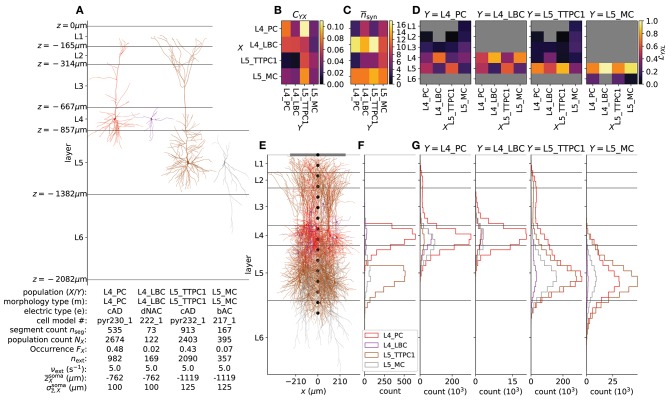
Details of the example network. **(A)** Biophysically detailed neuron models of the network, with depth-values of boundaries of layers 1–6. The lower left table summarizes population names (*X* – presynaptic; *Y* – postsynaptic) which here coincide with morphology type (m); electric type (e); cell model #; compartment count per single-cell model ($n_{j}^{\text{seg}}$); number of cells *N*_*X*_ in each population; occurrence *F*_*X*_ (defined as $N_{X}/\underset{X}{\sum }N_{X}$); the number of external synapses on each cell *n*_ext_; rate expectation of external synapses ν_ext_; the expected mean ${\bar{z}}_{X}^{\text{soma}}$ and standard deviation $\sigma_{\bar{z},X}^{\text{soma}}$ of the normal distribution N from which somatic depths are drawn. **(B)** Pairwise connection probability *C*_*YX*_ between cells in presynaptic populations *X* and postsynaptic populations *Y*. **(C)** Average number ${\bar{n}}^{\text{syn}}$ of synapses created per connection between *X* and *Y*. **(D)** Layer specificity of connections $L_{YXL}$ (Hagen et al., 2016) from each presynaptic population *X* onto each postsynaptic population *Y*. Gray values denote $L_{YXL}=0$. **(E)** Illustration of cylindrical geometry of populations including a laminar recording device for extracellular potentials (black circular markers) and a single ECoG electrode above layer 1 (gray line). *n* = 15 neurons of each population are shown in their respective locations. **(F)** Laminar distribution of somas for each network population (Δ*z* = 50 μm) in one instantiation of the circuit. **(G)** Laminar distribution of synapses across depth onto each postsynaptic population *Y* from presynaptic populations *X* (Δ*z* = 50 μm).

For simplicity of this demonstration, we here use only four different single-cell models as shown in Figure 2A for the different network populations. For layers 4 and 5 we chose the most common excitatory cell type and most common inhibitory interneuron cell type, in accordance with statistics of the reconstructed microcircuit of Markram et al. (2015) as provided on the NMC portal. The table in Figure 4A summarizes population names (*X*– presynaptic; *Y*– postsynaptic) which here coincide with morphology type (m), electric type (e), cell model #, compartment count per single-cell model ($n_{j}^{\text{seg}}$), number of cells *N*_*X*_ in each population, occurrence $F_{X}\equiv N_{X}/\underset{X}{\sum }N_{X}$, the number of external synapses on each cell *n*_ext_, rate expectation of external synapses ν_ext_ and the mean ${\bar{z}}_{X}^{\text{soma}}$ and standard deviation $\sigma_{\bar{z},X}^{\text{soma}}$ of the normal distribution $N\left({\bar{z}}_{X}^{\text{soma}},\sigma_{\bar{z},X}^{\text{soma}}\right)$ from which somatic depths are drawn for each population. The cell type can be derived from the “m” and “e” type in the table. Using the nomenclature of Markram et al. (2015), L4 and L5 are abbreviations for layer 4 and 5; PC – pyramidal cell; LBC – large basket cell; TTPC1 – thick-tufted pyramidal cell with a late bifurcating apical tuft; MC – Martinotti cell; cAD – continuous adapting; dNAC – delayed non-accommodating; bAC – burst accommodating. Thus, L4_PC_cAD corresponds to a layer 4 pyramidal cell with a continuously adapting firing pattern as a response to depolarizing step current and so forth. As multiple variations of the same cell types are provided on the NMC portal, the cell model # can be used to identify the particular single-cell model and corresponding file sets used in the network described here. These single-cell model files can be downloaded one after another from the portal as for example L5_TTPC1_cADpyr232_1.zip, or all together in a single archive. For simplicity we ignore heterogeneity in e-types for each m-type, thus the population counts *N*_*X*_ correspond to the count per m-type in the reconstructed microcircuit. Note for the present network description that {*X, Y*, m} ∈ {L4_PC, L4_LBC, L5_TTPC1, L5_MC}.

#### 2.5.2. Population Geometry

The centers of somatic compartments for all cells *i* ∈ *X* were distributed with even probability within a circular radius of 210 μm corresponding to the radius of the reconstructed somatosensory column in Markram et al. (2015). The corresponding depths were drawn from the normal distribution $N\left({\bar{z}}_{X}^{\text{soma}},\sigma_{\bar{z},X}^{\text{soma}}\right)$ using population-specific mean and standard deviations given in Figure 4A. Neuron positions resulting in any neuron compartments protruding above the hypothetical cortical surface at *z* = 0 or below layer 6 at *z* = −2082 μm were redrawn from the depth distribution. All cells were rotated around their local vertical *z*-axis by a random angle θ ∈ [0, 2π).

#### 2.5.3. Synapse Models

For synapses made by cells in a presynaptic population *X* onto a postsynaptic population *Y* we used synapse model files provided with the single-cell model files from the NMC portal. There are two base models with connection-specific parameterization which were obtained from the portal. Excitatory synapses are modeled as probabilistic AMPA and NMDA receptors, while inhibitory synapses are modeled as probabilistic GABA_A_ receptors. Both synapse types were modeled with presynaptic short-term plasticity. The synapse parameterization procedure and validation is described in detail in Markram et al. (2015), with code implementations based on Fuhrmann et al. (2002). The synapse parameters are summarized in Table 1, detailing the synapse model names, average synaptic conductances ${\bar{g}}^{\text{syn}}$ and corresponding standard deviations $\sigma_{\bar{g}}^{\text{syn}}$, release probabilities *P*_*u*_, relaxation time constants from depression τ_Dep_, relaxation time constants from facilitation τ_Fac_, ratios of NMDA vs. AMPA (excitatory connections only), rise and decay time constants $\tau_{U}^{\text{r}}$ and $\tau_{U}^{\text{d}}$ of the two-exponential conductances of each current type *U* ∈ {AMPA, NMDA, GABAA}, and reversal potentials *e*^syn^. Random conductances for each individual synapse were drawn from the capped normal distribution $N\left({\bar{g}}^{\text{syn}},\sigma_{\bar{g}}^{\text{syn}}\right)H\left(g-g_{\text{min}}\right)$. For our network we set the minimum synaptic conductance to be *g*_min_ = 0 nS.

**Table 1:** Summary of intrinsic synapse parameters.

		**Postsynaptic population** (***Y***)				
		**Parameter**	**L4_PC**	**L4_LBC**	**L5_TTPC1**	**L5_MC**
presynaptic population (*X*)	L4_PC	syn. model ${\bar{g}}^{\text{syn}}$ (nS) $\sigma_{\bar{g}}^{\text{syn}}$ (nS) *P*_*u*_ τ_Dep_ (ms) τ_Fac_ (ms) NMDA ratio $\tau_{\text{AMPA}}^{\text{r}}$ (ms) $\tau_{\text{AMPA}}^{\text{d}}$ (ms) $\tau_{\text{NMDA}}^{\text{r}}$ (ms) $\tau_{\text{NMDA}}^{\text{d}}$ (ms) *e*^syn^ (mV)	ProbAMPANMDA 0.3 0.11 0.859 670 17 0.4 0.2 1.737 0.29 43 0	ProbAMPANMDA 0.3 0.11 0.398 560 130 0.4 0.2 1.74 0.29 43 0	ProbAMPANMDA 0.3 0.11 0.5 670 17 0.4 0.2 1.742 0.29 43 0	ProbAMPANMDA 0.3 0.11 0.093 140 660 0.4 0.2 1.742 0.29 43 0
	L4_LBC	syn. model ${\bar{g}}^{\text{syn}}$ (nS) $\sigma_{\bar{g}}^{\text{syn}}$ (nS) *P*_*u*_ τ_Dep_ (ms) τ_Fac_ (ms) $\tau_{\text{GABAA}}^{\text{r}}$ (ms) $\tau_{\text{GABAA}}^{\text{d}}$ (ms) *e*^syn^ (mV)	ProbGABAAB 0.89 1.3 0.213 730 21 0.2 7.604 -80	ProbGABAAB 0.33 0.15 0.254 700 21 0.2 8.373 -80	ProbGABAAB 0.98 1.3 0.226 750 21 0.2 7.364 -80	ProbGABAAB 0.33 0.16 0.253 710 21 0.2 8.349 -80
	L5_TTPC1	syn. model ${\bar{g}}^{\text{syn}}$ (nS) $\sigma_{\bar{g}}^{\text{syn}}$ (nS) *P*_*u*_ τ_Dep_ (ms) τ_Fac_ (ms) NMDA ratio $\tau_{\text{AMPA}}^{\text{r}}$ (ms) $\tau_{\text{AMPA}}^{\text{d}}$ (ms) $\tau_{\text{NMDA}}^{\text{r}}$ (ms) $\tau_{\text{NMDA}}^{\text{d}}$ (ms) *e*^syn^ (mV)	ProbAMPANMDA 0.29 0.11 0.5 670 17 0.4 0.2 1.743 0.29 43 0	ProbAMPANMDA 0.3 0.11 0.369 550 140 0.4 0.2 1.743 0.29 43 0	ProbAMPANMDA 0.31 0.11 0.5 670 17 0.4 0.2 1.744 0.29 43 0	ProbAMPANMDA 0.3 0.11 0.092 150 690 0.4 0.2 1.741 0.29 43 0
	L5_MC	syn. model ${\bar{g}}^{\text{syn}}$ (nS) $\sigma_{\bar{g}}^{\text{syn}}$ (nS) *P*_*u*_ τ_Dep_ (ms) τ_Fac_ (ms) $\tau_{\text{GABAA}}^{\text{r}}$ (ms) $\tau_{\text{GABAA}}^{\text{d}}$ (ms) *e*^syn^ (mV)	ProbGABAAB 0.66 0.15 0.3 1200 2.1 0.2 8.291 -80	ProbGABAAB 0.33 0.15 0.25 700 21 0.2 8.295 -80	ProbGABAAB 0.66 0.15 0.299 1200 2.2 0.2 8.271 -80	ProbGABAAB 0.33 0.15 0.252 710 21 0.2 8.339 -80

#### 2.5.4. Extrinsic Input

Synapses from external inputs to the neurons in our network were modeled similarly to excitatory synapses of intrinsic network connections. For inputs to a population *Y* in layer *L* we chose to duplicate the synapse parameters of connections made by the presynaptic excitatory population within the same layer (as we were unable to assess what parameters were used for extrinsic connections in Markram et al., 2015). Our synapse parameters are given in Table 2. For each cell in the network we created *n*_ext_ synapses set randomly onto dendritic and apical compartments with compartment specificity of connections $S_{jn}/\underset{n\in \left\{\text{dend},\text{apic}\right\}}{\sum }S_{jn}$, where *S*_*jn*_ denotes surface area of compartment *n* of cell *j*. The random activation times of each synapse were set using Poisson processes with rate expectation ν_ext_ for the duration of the simulation. The values for *n*_ext_ and ν_ext_ are given in Figure 4A, and were set by hand in order to maintain spiking activity in all populations.

**Table 2:** Synapse parameters for extrinsic input.

		**Postsynaptic population** (***Y***)				
		**Parameter**	**L4_PC**	**L4_LBC**	**L5_TTPC1**	**L5_MC**
Presynaptic pop. (*X*)	ext	syn. model ${\bar{g}}^{\text{syn}}$ (nS) $\sigma_{\bar{g}}^{\text{syn}}$ (nS) *P*_*u*_ τ_Dep_ (ms) τ_Fac_ (ms) NMDA ratio $\tau_{\text{AMPA}}^{\text{r}}$ (ms) $\tau_{\text{AMPA}}^{\text{d}}$ (ms) $\tau_{\text{NMDA}}^{\text{r}}$ (ms) $\tau_{\text{NMDA}}^{\text{d}}$ (ms) *e*^syn^ (mV)	ProbAMPANMDA 0.3 0.11 0.859 670 17 0.4 0.2 8.291 0.29 43 0	ProbAMPANMDA 0.33 0.15 0.254 700 21 0.4 0.2 8.295 0.29 43 0	ProbAMPANMDA 0.31 0.11 0.5 670 17 0.4 0.2 8.271 0.29 43 0	ProbAMPANMDA 0.33 0.15 0.252 710 21 0.4 0.2 8.339 0.29 43 0

#### 2.5.5. Connectivity Model

Random connections in our network were set using the Python-implementation of the “connection-set algebra” of Djurfeldt (2012) and Djurfeldt et al. (2014) (github.com/INCF/csa). Using this formalism, we constructed boolean connectivity matrices ${\text{C}}_{YX}^{\left(r\right)}$ for postsynaptic cells *j*^(*r*)^⊂*Y* distributed across each separate parallel MPI rank (denoted by the superset “(*r*)” for rank number) and presynaptic cells *i* ∈ *X*. Each instance of ${\text{C}}_{YX}^{\left(r\right)}$ had shape $\left(N_{X}\times N_{j^{\left(r\right)}\subset Y}\right)$, with entries equal to True denoting connections from cell *i* to *j*^(*r*)^, as expressed mathematically by

(19) $$\begin{array}{l} {\text{C}}_{YX}^{\left(r\right)}\left(C_{YX}\right)\left(i,j^{\left(r\right)}\right)=\{\begin{array}{l} \text{True with probability }C_{YX}\;,\\ \text{False otherwise }. \end{array} \end{array}$$

For *X* = *Y* and *i* = *j*^(*r*)^, entries in ${\text{C}}_{YX}^{\left(r\right)}$ were set to False (no autapses). We used fixed connection probabilities *C*_*YX*_ as obtained from the NMC portal between our chosen m-types.

#### 2.5.6. Multapses

As multiple synapses per connection appear to be a prominent feature in cortical networks (see Markram et al., 2015; Reimann et al., 2015 and references therein), we drew for every connection between presynaptic cell *i* and postsynaptic cell *j* a random number of synapses *n*^syn^ rounded to an integer from the capped normal distribution $N\left({\bar{n}}^{\text{syn}},\sigma_{\bar{n}}^{\text{syn}}\right)H\left(n\right)$. Conduction delays from action-potential detection (threshold θ_AP_ = −10 mV) in cell *i* for each corresponding synapse onto cell *j* were drawn from the distribution $N\left({\bar{\delta }}^{\text{syn}},\sigma_{\bar{\delta }}^{\text{syn}}\right)H\left(\delta -\delta_{\text{min}}\right)$. For our network we set the minimum delay δ_min_ = 0.3 ms for all connections.

#### 2.5.7. Layer-specificity of connections

In order to position each individual synapse of a connection on a cell *j* ∈ *Y*, in a simplified manner that depended on the degree of overlap between presynaptic axons and postsynaptic dendrites (“Peter's rule”), we calculated for each postsynaptic population *Y* layer-specificities of connections $L_{YXL}$ in layer *L* for synapses made by presynaptic populations *X* (Hagen et al., 2016), by first computing the sums $\Delta s_{iXL}=\underset{n\in \text{axon}}{\sum }\Delta s_{inXL}$, that is, the total axon length of a presynaptic cell type per layer *L* and sums $\Delta s_{jYL}=\underset{n\in \left\{\text{soma},\text{dend}\right\}}{\sum }\Delta s_{jnYL}$ of total dendrite and soma length for each postsynaptic cell type across each layer. Then we defined the layer-specificity of connections as

$$\begin{array}{l} L_{YXL}=\sqrt{\Delta s_{iXL}\Delta s_{jYL}}/\underset{L}{\sum }\sqrt{\Delta s_{iXL}\Delta s_{jYL}}\;. \end{array}$$

The sums $\underset{L}{\sum }$ $L_{YXL}$ = 1 for all *X* and *Y*. Synapse sites of connections onto cell *j* were then set randomly with a compartment specificity of connections $S_{jn}\underset{L}{\sum }{\text{Pr}}_{N\left(L_{YXL},\Delta \text{L}/2\right)}\left(z_{nj}\right)/\underset{n}{\sum }S_{jn}$, where *S*_*jn*_ is the surface area of compartment *n* of the cell *j* centered at depth *z*_*nj*_ and ${\text{Pr}}_{N}\left(\ldots \right)$ the probability density function of the distribution $N\left(L_{YXL},\Delta L/2\right)$. Δ*L* denotes the thickness of layer *L*.

All connectivity parameter values ($C_{YX},{\bar{n}}^{\text{syn}}$, $\sigma_{\bar{n}}^{\text{syn}}$, ${\bar{\delta }}^{\text{syn}},\sigma_{\bar{\delta }}^{\text{syn}},L_{YXL}$) are summarized in Table 3. Visual representations of $C_{YX},{\bar{n}}^{\text{syn}}$ and $L_{YXL}$ are shown in Figures 4B–D. Figure 4E shows 15 cells in each population *X* with corresponding distribution of *N*_*X*_ somas across depth (Δ*z* = 50 μm) in Figure 4F. Panel G shows the resulting distribution of synapses across depth for all combinations of *Y* and *X* (Δ*z* = 50 μm).

**Table 3:** Summary of connectivity parameters.

		**Postsynaptic population** (***Y***)				
		**Parameter**	**L4_PC**	**L4_LBC**	**L5_TTPC1**	**L5_MC**
Presynaptic population (*X*)	L4_PC	*C*_*YX*_ ${\bar{n}}_{\text{syn}}$ $\sigma_{\bar{n},\text{syn}}$ ${\bar{\delta }}_{\text{syn}}$ (ms) $\sigma_{\bar{\delta },\text{syn}}$ (ms) $L_{YX\text{L}1}$ $L_{YX\text{L}2}$ $L_{YX\text{L}3}$ $L_{YX\text{L}4}$ $L_{YX\text{L}5}$ $L_{YX\text{L}6}$	0.076 3.3 1.4 1.35 0.867 0.0 0.058 0.152 0.336 0.454 0.0	0.042 7.9 3.0 1.17 0.763 0.0 0.0 0.0 0.53 0.47 0.0	0.11 4.3 1.7 1.433 0.817 0.0 0.069 0.106 0.105 0.719 0.0	0.034 7.6 2.7 1.521 0.978 0.0 0.0 0.0 0.0 0.73 0.27
	L4_LBC	*C*_*YX*_ ${\bar{n}}_{\text{syn}}$ $\sigma_{\bar{n},\text{syn}}$ ${\bar{\delta }}_{\text{syn}}$ (ms) $\sigma_{\bar{\delta },\text{syn}}$ (ms) $L_{YX\text{L}1}$ $L_{YX\text{L}2}$ $L_{YX\text{L}3}$ $L_{YX\text{L}4}$ $L_{YX\text{L}5}$ $L_{YX\text{L}6}$	0.063 16.0 6.2 1.006 0.367 0.0 0.0 0.1 0.672 0.228 0.0	0.062 14.0 6.0 1.076 0.395 0.0 0.0 0.0 0.818 0.182 0.0	0.056 17.0 7.2 1.064 0.399 0.0 0.0 0.109 0.328 0.563 0.0	0.027 10.0 3.5 1.677 0.494 0.0 0.0 0.0 0.0 1.0 0.0
	L5_TTPC1	*C*_*YX*_ ${\bar{n}}_{\text{syn}}$ $\sigma_{\bar{n},\text{syn}}$ ${\bar{\delta }}_{\text{syn}}$ (ms) $\sigma_{\bar{\delta },\text{syn}}$ (ms) $L_{YX\text{L}1}$ $L_{YX\text{L}2}$ $L_{YX\text{L}3}$ $L_{YX\text{L}4}$ $L_{YX\text{L}5}$ $L_{YX\text{L}6}$	0.011 2.5 0.89 2.374 0.811 0.0 0.02 0.129 0.244 0.608 0.0	0.0069 6.1 2.1 2.227 0.903 0.0 0.0 0.0 0.379 0.621 0.0	0.063 6.2 2.6 1.445 0.653 0.0 0.02 0.078 0.066 0.836 0.0	0.045 9.2 3.1 1.372 0.577 0.0 0.0 0.0 0.0 0.739 0.261
	L5_MC	*C*_*YX*_ ${\bar{n}}_{\text{syn}}$ $\sigma_{\bar{n},\text{syn}}$ ${\bar{\delta }}_{\text{syn}}$ (ms) $\sigma_{\bar{\delta },\text{syn}}$ (ms) $L_{YX\text{L}1}$ $L_{YX\text{L}2}$ $L_{YX\text{L}3}$ $L_{YX\text{L}4}$ $L_{YX\text{L}5}$ $L_{YX\text{L}6}$	0.04 12.0 3.9 1.91 0.994 0.111 0.13 0.249 0.329 0.18 0.0	0.035 12.0 3.7 1.732 0.663 0.0 0.0 0.0 0.735 0.265 0.0	0.083 14.0 5.6 2.252 1.549 0.136 0.187 0.209 0.124 0.344 0.0	0.038 12.0 3.4 1.341 0.787 0.0 0.0 0.0 0.0 0.926 0.074

#### 2.5.8. Computation of Extracellular Potentials Inside Cortical Column

For our multicompartment neuron network we chose to compute the extracellular potential vertically through the center of the column, with the most superficial contact at the top of layer 1 (*z* = 0) to a depth of *z* = −1500 μm within layer 6. The inter-contact distance was Δ*z* = 100 μm, and contacts were assumed to be circular with radius *r*_contact_ = 5 μm and surface normal vectors aligned with the horizontal *y*-axis. For the electrode surface averaging we used *m* = 50 (cf. Equation 8 and Lindén et al., 2014). For the calculation of extracellular potential inside the cortical column we assumed a homogeneous, isotropic, linear and ohmic extracellular conductivity σ_e_ = 0.3 S/m.

#### 2.5.9. Computation of ECoG Signal From Method-of-Images

The extracellular potential on top of cortex (ECoG) was computed by means of the Method-of-Images (MOI, see section 2.2.2). In the example, the conductivity below the contact was set as σ_*G*_ = σ_*T*_ = 0.3 S/m, corresponding to the gray-matter value used above, while the conductivity on top of cortex was to set to be fully insulating, that is, σ_*T*_ = 0 S/m. This could correspond to the situation where a grid of ECoG contacts are embedded in an insulating material (see for example, Castagnola et al., 2014). We further considered a single circular ECoG disk electrode with contact radius *r* = 250 μm with its surface normal vector perpendicular to the brain surface. The disk electrode was centered at the vertical population axis and positioned at the upper boundary of layer 1. For the disk-electrode approximation (cf. Equation 8) we set *m* = 500. (Note that the present MoI implementation requires all transmembrane currents to be represented as point sources confined within the boundaries of the middle (cortical) layer.

#### 2.5.10. Computation of EEG and MEG Signals

The most direct approach for computing EEG and MEG signals would be to (i) compute the per-neuron current dipole moment, (ii) compute the contribution to the signals from each neuron, and (iii) sum these signals to get the total EEG and MEG signals from the entire network. To reduce the computational demands, we instead compute the per-population current dipole moment **p**_*X*_(*t*) using equation 12. The total current dipole moment is then obtained by summing over all populations, that is, $\text{p}=\underset{X}{\sum }{\text{p}}_{X}$.

From **p**_*X*_ we computed the EEG (surface electric potentials on the scalp layer) of the four-sphere head model as described above, and similarly magnetic fields **B**_**p**_. For the four-sphere head model we assumed conductivities σ_*s*_ ∈ {0.3, 1.5, 0.015, 0.3} S/m and radii *r*_*s*_ ∈ {79, 80, 85, 90} mm for brain, cerebrospinal fluid (CSF), skull and scalp, respectively (Nunez and Srinivasan, 2006; Næss et al., 2017). We positioned each population current dipole **p**_*X*_ below the brain-CSF boundary on the vertical *z*-axis (thus *x* = *y* = 0) at $z=r_{1}+{\bar{z}}_{X}^{\text{soma}}$, where ${\bar{z}}_{X}^{\text{soma}}$ was the average soma depth within each population. Surface potentials, that is, EEG potentials, and magnetic fields where computed for polar angles θ ∈ [−π/4, π/4] with angular resolution Δθ = π/16 as illustrated in Figure 2D (azimuth angles φ = 0), resulting in a contact separation along the arc of *r*_4_π/16 ≈ 18 mm. Different magnetoelectroencephalogram (MEG) equipment may be sensitive to different components of the magnetic field (Hämäläinen et al., 1993). We show different scalar components of the magnetic field computed on the surface of the four-sphere head model as described above (in section 2.3.5).

#### 2.5.11. Simulation Details

Simulations were run for a total duration of *T* = 1, 500 ms with a simulation step size *dt* = 0.0625 ms (16 kHz sampling frequency). The first 500 ms were discarded as startup transient. All neurons were initialized at a membrane voltage $V_{\text{init}}^{\text{m}}=-77\;$mV and temperature *T*_celsius_ = 34°C (affecting membrane-channel dynamics).

### 2.6. Technical Details

#### 2.6.1. Code Availability

All source codes and development history of past and present versions of LFPy are publicly available on GitHub (see github.com/LFPy/LFPy), using “git” (git-scm.com) for code provenance tracking. LFPy is released with an open-source software licence (GPL), which alongside GitHub functionality for listing issues, integration with automated testing, easy forking, local development and merges of upstream changes, facilitates continued, community-based LFPy development.

#### 2.6.2. Requirements

LFPy 2.0 requires Python (continuously tested w. v2.7, v3.4-3.6), an MPI (message-parsing interface) implementation such as OpenMPI, NEURON v7.4 or newer compiled with MPI and bindings for Python, Cython, and the Python packages mpi4py, numpy, scipy, h5py, csa (github.com/INCF/csa) and NeuroTools (neuralensemble.org/NeuroTools). In order to run all example files also matplotlib and Jupyter (jupyter.org) have to be installed, but prebuilt Python distributions such as Anaconda (anaconda.com) should provide these common Python packages, or easy means of installing LFPy dependencies (issuing, for example, "conda install mpi4py" on the command line). Detailed instructions for installing dependencies for common operating systems (MacOS, Linux, Windows) are provided in the online LFPy documentation (lfpy.readthedocs.io).

#### 2.6.3. Installation

The latest stable LFPy release on the Python Package Index (pypi.python.org) can be installed by issuing:

$ pip install LFPy --user

which may prompt the install of also other missing dependencies. The command

$ pip install --upgrade --no-deps LFPy --user

may be used to upgrade an already existing installation of LFPy (without upgrading other dependencies). In order to obtain all LFPy source codes and corresponding example files, we recommend users to checkout the LFPy source code on GitHub, after installing the git version control software:

$ **cd** <path to repository folder>
$ git clone https://github.com/LFPy/LFPy.git
$ **cd** LFPy
$ pip install -r requirements --user
$ python setup.py develop --user

More detail is provided on lfpy.readthedocs.io.

#### 2.6.4. Reproducibility

The simulated results and analysis presented here were made possible using Python 2.7.11 with the Intel(R) MPI Library v5.1.3, NEURON v7.5 (1472:078b74551227), Cython v0.23.4, LFPy (github.com/LFPy/LFPy, SHA:0d1509), mpi4py v2.0.0, numpy v1.10.4, scipy v0.17.0, h5py v2.6.0, parameters (github.com/NeuralEnsemble/parameters, SHA:v0aaeb), csa (github.com/INCF/csa, SHA:452a35) and matplotlib v2.1.0 running in parallel using 120-4800 cores on the JURECA cluster in Jülich, Germany, composed of two 2.5 GHz Intel Xeon E5-2680 v3 Haswell CPUs per node (2 x 12 cores), running the CentOS 7 Linux operating system. Each node had at least 128 GB of 2133 MHz DDR4 memory. All software packages were compiled using the GNU Compiler Collection (GCC) v4.9.3. All source codes for this study are provided as LFPy example files on GitHub.

## 3. Results

### 3.1. Single-Neuron Activity and Extracellular Measurements

The first version of LFPy (Lindén et al., 2014) assumed the model neurons to be embedded in an infinite homogeneous volume conductor and was most suited to compute extracellular potentials (spikes, LFPs) inside the brain. One new feature of LFPy 2.0 compared to the first version of LFPy is that electrical potentials outside cortex (ECoG, EEG), as well as magnetic fields both inside and outside cortex (MEG), can be computed. These new measures are illustrated in Figure 2 for a single synaptically activated “pyramidal” neuron (composed of soma and dendrite sections only).

Figure 2A presents a basic LFPy simulation example where a passive neuron model with simplified morphology receives a single synaptic input current (inset I). We computed the extracellular potential in the *xz*-plane (color image plot), using the assumption of line sources for each dendritic compartment, a spherical current source representing the soma, and homogeneous conductivity (7). The postsynaptic response is reflected as a somatic depolarization (inset II) and as a deflection in the extracellular potential in the location **r** (blue dot, inset III). The corresponding current dipole moment **p**(**r**, *t*) was computed using equation 12 and is illustrated by the black arrow. The *x*- and *z*-components ($\text{p}\cdot \hat{\text{x}},\text{p}\cdot \hat{\text{z}}$) of the current dipole moment are illustrated in inset IV, and we note the much larger dipole moment component in the vertical *z*-direction compared to the lateral *x*-direction. We do not show the *y*-component of the current dipole moment as all segments in this simplified neuronal morphology are located in the *xz*-plane (hence $\text{p}\cdot \hat{\text{y}}=0$).

To illustrate the fact that a current dipole potential (Equation 15) gives a good approximation to the extracellular potential ϕ far away from the neuron, we compare with results from using the more comprehensive line-source method (Equation 6) in Figure 2B: The line-source potential ϕ is shown inside the dashed circle of radius *r* = 500 μm, while the dipole potential ϕ_**p**_ is shown outside the circle. The inset shows the dipole potential corresponding to the colored dots located at a distance of 750 μm.

In Figure 2C we similarly compute the magnetic field for radii *r* > 500 μm using the current dipole moment (Equation 16), and axial currents inside (Equation 18). The axial currents were computed from per-compartment membrane potentials as described in section 2.1.2. For both color image plot and the inset, we show the dominating magnetic field component, that is, the *y*-component. As for the electrical potential in Figure 2B, we see that the predicted magnetic fields match well at the *r* = 500 μm interface.

Figure 2D illustrates the layout of scalp-layer measurement sites on the four-sphere head model described in section 2.3.3. The numbered points along the outer scalp layer represents measurement locations for EEG and MEG signals. The single current dipole moment is positioned beneath the CSF-brain boundary on the vertical *z*-axis (see caption for details). Figure 2E shows the corresponding scalp surface potentials which is dominated by the *z*-component of the current dipole moment ($\text{p}\cdot \hat{\text{z}}$, Figure 2A inset IV). Figure 2F shows the corresponding dominant azimuthal tangential magnetic field component (${\text{B}}_{p}\cdot \hat{\varphi }$) computed from the current dipole moment using equation 16. At the center location (location 5) only the *x*-component ($\text{p}\cdot \hat{\text{x}}$) contributes to the signal, in the other locations both the *x*- and *y*-components contribute.

### 3.2. Network Activity and Extracellular Measurements

The second main new feature of LFPy 2.0 is the possibility to simulate recurrently connected networks of neurons in parallel. Our exemple network, shown in Figure 4, demonstrating this new feature is based on a subset of cortical single-cell models, synapse models and connectivity data from Markram et al. (2015) obtained from The Neocortical Microcircuit Collaboration (NMC) Portal (Ramaswamy et al., 2015). The implementation is described in detail in section 2.5.

In addition to supporting simulations of neuronal networks with simplified or biophysically detailed single-neuron models in parallel, LFPy 2.0 allows for concurrent calculations of extracellular measures of network activity. Specifically, the extracellular potentials at specific positions can be computed at each time step which avoids the memory-demanding process of recording transmembrane currents in all compartments for the duration of the simulation, either to disk or to memory. In the present example, the current dipole moment was calculated at every time step, and this amounted to a useful dimensionality reduction, as only the *x, y, z*-axis components of **p** per population *X* had to be stored. Assuming serial execution, then for each neuron population *X*, the total memory consumption is then reduced by a factor $3/\left(N_{X}n^{\text{seg}}\right)$ where *N*_*X*_ is the population size and *n*^seg^ the number of compartments per neuron (see Figure 4A for values), compared to storing currents. The per-population current dipole moments were then used to predict EEG scalp surface potentials and MEG signals in the corresponding locations. Note that per-population current dipole moments can be stored, EEG and MEG signal can be computed with other head models at a later stage.

#### 3.2.1. Network Spiking Activity

**Figure 5:**
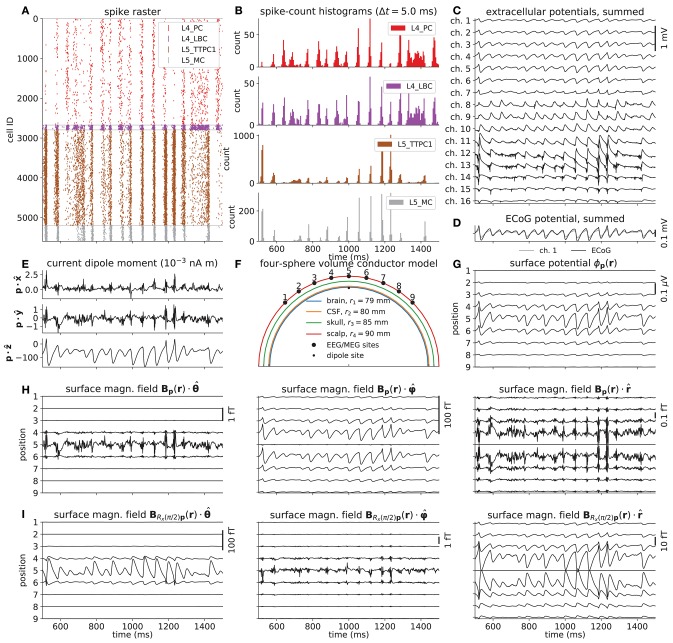
Intra- and extracellular measures of activity in example network. **(A)** Spike raster plot for each population. Each row of dots corresponds to the spike train of one neuron, color coded by population. **(B)** Population spike rates computed by summing number of spike events in each population in temporal bins of width Δ*t* = 5 ms. **(C)** Extracellular potentials as function of depth assuming an infinite volume conductor. **(D)** Extracellular potential on top of cortex (ECoG) assuming a discontinuous jump in conductivity between brain (σ = 0.3 S/m) and a non-conducting cover medium (σ = 0 S/m) and electrode surface radius *r* = 250 μm. The signal is compared to the channel 1 extracellular potential in **(C)** (gray line). **(E)** Component-wise contributions to the total current dipole moment **p**(*t*) summed over population contributions. **(F)** Illustration of upper half of the four-sphere head model (with conductivities σ_*s*_ ∈ {0.3, 1.5, 0.015, 0.3} S/m and radii *r*_*s*_ ∈ {79, 80, 85, 90} mm for brain, csf, skull and scalp, respectively), dipole location in inner brain sphere and scalp measurement locations. The sites in the *xz*-plane numbered 1–9 mark the locations where electric potentials and magnetic fields are computed, each offset by an arc length of *r*_4_π/16 ≈ 18 mm. **(G)** EEG scalp potentials from multicompartment-neuron network activity with radially oriented populations. **(H)** Tangential and radial components of the head-surface magnetic field (MEG) from multicompartment-neuron network activity with radially oriented population. **(I)** Tangential and radial components of the magnetic field (MEG) on the head surface, with underlying dipole sources rotated by an angle θ = π/2 around the *x*-axis (thus with apical dendrites pointing into the plane). (Note that at position 5, the unit vectors $\hat{\varphi }$ and $\hat{\theta }$ are defined to be directed in the positive *y*- and *x*-directions, respectively).

Figure 5 shows the various predicted measurements for a one-second period of network activity. The spike raster and corresponding spike-count histogram (Figures 5A,B) demonstrate the network's tendency to produce synchronous irregular patterns of activity with the parameterization summarized in section 2.5, Tables 1–3 and Figure 4. The per-neuron spike occurrences in the excitatory populations L4_PC and L5_TTPC1 were sparser than for the inhibitory populations L4_LBC and L5_MC. As in the full circuit of Markram et al. (2015), it is possible that an asynchronous network state could have been obtained by modifying extracellular [Ca^2+^]_o_-dependent release probabilities *P*_*u*_ for the different synapse types in the model (Borst, 2010; Markram et al., 2015). A modification of release probabilities can shift the effective balance between excitatory and inhibitory synapse activations, but also incorporation of a larger sample of heterogeneous cell types in the model could have brought the network into an asynchronous state, essentially by increasing the amount of inhibitory feedback. In particular interneuron expression in neocortex is known to be more heterogeneous and more dense than demonstrated here (Markram et al., 2004, 2015). However, as our main focus here is to present new simulation technology now incorporated in LFPy, we did not pursue this line of inquiry.

#### 3.2.2. Local Field Potentials (LFPs)

The extracellular potentials as would be measured by a 16-channel laminar probe positioned through the center axis of the cylindrical column, are shown in Figure 5C. The computed extracellular potentials are observed to be of the same order of magnitude as experimentally measured spontaneous potentials (≃0.1–1 mV, see Maier et al., 2010; Hagen et al., 2015; Reyes-Puerta et al., 2016). We further observe that the synchronous events seen in the spiking activity (Figure 5A) are reflected as substantial fluctuations in the extracellular potential with amplitudes close to 0.5 mV.

The signals in neighboring channels are further observed to be fairly correlated with comparable amplitudes, irrespective of the presence of somatic compartments at the depths of the contacts (Figure 4F). At the superficial channels 1–6, deflections in the electric potential following synchronous network activation are predominantly negative, while a change in sign occur around channel 7 (near the boundary between layer 3 and 4). The strongest deflections of the extracellular potential are typically observed at contacts within layer 5 (ch. 11–13), that is, at depths corresponding to the dense branching of basal dendrites and somas of the large layer 5 pyramidal neuron population. These deflections reflect that the soma compartments and basal dendrites are expected to act as dominant sources of the transmembrane currents setting up the extracellular potential (Lindén et al., 2010). Adding further to this, layers 4 and 5 also had the highest overall densities of excitatory and inhibitory synapses in the present model (Figure 4G). Some spike events (extracellular signatures of action potentials) are seen in ch. 15, produced by one or several neurons located near the virtual recording device.

**Figure 6:**
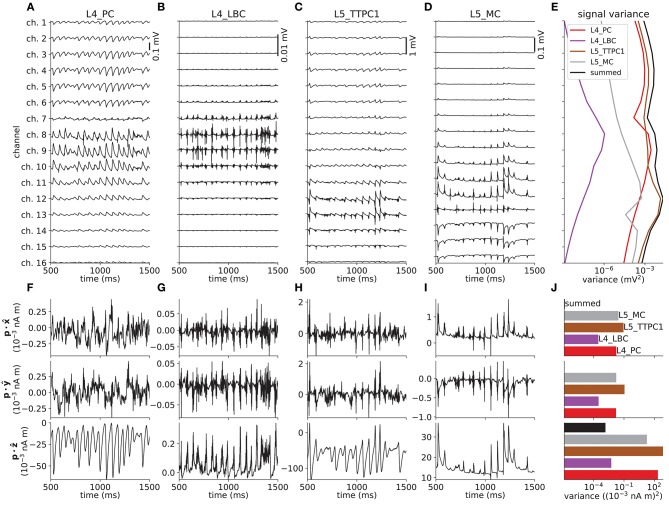
Per-population contributions to the extracellular potential and current dipole moment and corresponding signal variance. **(A–D)** Contributions to the extracellular potential from populations *X* ∈ {L4_PC, L4_LBC, L5_TTPC1, L5_MC} in the network across depth. **(E)** Extracellular potential variance across depth for contributions of each population, and for the sum over populations. **(F–I)** *x, y, z*-components of the per-population contribution to the summed current dipole moment. **(J)** Per-component current dipole moment variance for each population and for summed signals.

Further investigation of the different contributors (Figures 6A–D) to the extracellular potential (Figure 5C), revealed that most of the signal variance across depth can be explained by transmembrane currents of the two excitatory populations (Figure 6E). Even if the cell numbers in the two pyramidal-cell population were similar, population L5_TTPC1 contributed more to the signal than population L4_PC at all channels except at channels 8-9 (around which the L4_PC somas are positioned).

#### 3.2.3. ECoG Signal

Figure 5D compares the extracellular potential in the topmost channel 1 (gray line), predicted under the assumptions of dendritic line sources, somatic spherical sources and an infinite homogeneous extracellular medium (cf. Equation 7), with our ECoG prediction at the same depth (black line). The ECoG signal was computed assuming a wide contact (*r*_contact_ = 250 μm) aligned horizontally on top of a flat cortex (*z* = 0). Further, for the ECoG signal the method-of-images (MoI; cf. Equation 11) was used to account for a conductivity discontinuity at the cortical surface. Here, zero conductivity (mimicking, for example, the situation with an insulating mat surrounding the ECoG contact, Castagnola et al., 2014) was assumed above the cortical surface, while the gray-matter value of σ_e_ = 0.3 S/m was assumed below.

The amplitude of the ECoG trace was slightly increased compared to the potential measured by the smaller electrode. This amplitude increase can be attributed to the fact that a reduction in conductivity above the boundary would decrease the value of the denominator of equation 11, and hence increase the signal amplitude below insulating cortical surfaces (Pettersen et al., 2006). The expected increased signal amplitude from this conductivity step is here counter-measured by the larger diameter of the ECoG contact (*r*_contact_ = 250 μm vs. *r*_contact_ = 5 μm) resulting in an increased average distance from the signal source to the contact point averaged over the contact's surface. Detailed investigation of each signal normalized to the same standard deviation (not shown) revealed virtually indistinguishable features across time and in their power spectra.

#### 3.2.4. Current Dipole Moments

Figure 5E shows the three components of the total current dipole moment **p** stemming from the network activity. The most striking feature is the much larger *z*-component compared to the lateral *x*- and *y*-components. This large difference in component size, about two orders for magnitude, reflects (i) that the vertically aligned pyramidal cell morphologies span across several layers, and (ii) the near rotational symmetry of the model populations around the *z*-axis. Unlike the *z*-component, the lateral components largely cancel out. In the same way as for the extracellular potential, the two pyramidal populations are also the dominant sources of the total dipole moment (Figures 6F–J). We also note that the *z*-component of the population current dipole moment generally dominates the other components of the population dipoles, with the exception of the L4_LBC population. Here all components are tiny, reflecting the stellate dendritic morphology and the evenly distributed synapses onto the neurons in this population.

For our model network we note that the maximum magnitude of the current dipole moment is about 0.1 nAm, which is about two orders of magnitude smaller than previously estimated typical “mesoscopic” dipole strengths (Hämäläinen et al., 1993, p. 418).

#### 3.2.5. EEG Signals

As a demonstration of predicting non-invasive electric (“EEG”) signals outside of the brain with LFPy 2.0, we utilized the four-sphere head model (as implemented in class FourSphereVolumeConductor, see 2) and defined scalp-layer measurement locations as illustrated in Figure 5F. We assumed the modeled network to represent a piece of cortical network positioned at the top of a cortical gyrus, so that the population axes were in the radial direction of the spherical head model. The current dipoles (computed above) were positioned below the interface between the CSF and the brain, more specifically the layer-4 and layer-5 population dipoles were positioned at the depth of the center of layer 4 and layer 5, respectively.

As observed in Figure 5G, the temporal form of the scalp potentials corresponds directly to the temporal form of the dominant *z*-component of the current dipole moment in Figure 5E. For an infinite volume conductor it follows directly from 15 that the recorded scalp potential will be proportional to this dipole moment at recording positions directly (radially) above the dipole location. Likewise, inspection of the formulas for the four-sphere head model shows that this is also the case for the scalp-potential contributions from both the radial (Næss et al., 2017, Equations 5–6) and tangential (Næss et al., 2017, Equations 17–18) dipole components (although with different proportionality constants for the two components).

For the present example network comprising 5,594 neurons of which 5,077 are pyramidal cells, we observe the magnitudes of the fluctuating scalp potential directly on top of the dipole sites to be on the order of 0.1 μV. This is about two orders of magnitude smaller than the typical size of measured EEG signals of ~10 μV (Nunez and Srinivasan, 2006, Figure 1.1).

The weakly conducting skull layer (compared to the highly conductive brain, spinal fluid and scalp layers) results in a spatial “low-pass filter effect” from volume conduction (Nunez and Srinivasan, 2006, Ch. 6). This low-pass effect accounts for the relatively weak attenuation of the EEG signal with lateral distance from the center position (position 5 in Figure 5F) along the head surface, as observed in Figure 5G. On the surface of a spherical volume conductor with homogeneous conductivity inside the sphere, but otherwise zero conductivity outside the sphere's surface (1-sphere head model), the potential from a current dipole would decay in amplitude at a higher rate compared to our 4-sphere head-model case with a spherical skull layer with low conductivity. However, in an infinite homogeneous volume conductor the decay in electric potential along the putative sphere's surface would decay with a lower rate than both the 1-sphere and 4-sphere head models, see Nunez and Srinivasan (2006, Ch. 6) for a comparison.

#### 3.2.6. MEG Signals

The computed current dipole moments in Figure 5E was also used to compute MEG signals. Figure 5H shows the computed magnetic fields for the same set-up providing the EEG signals in Figure 5G, that is, radially oriented population current dipoles. In this situation the only sizable magnetic field is directed in the tangential direction around the vertical *z*-axis. With our spherical coordinates this corresponds to the **φ**-direction where the unit vector $\hat{\varphi }$ points in counter-clockwise direction. Note also that the magnetic field is almost zero straight above the dipole (position 5), as here the vectors **p** and **R** are near parallel so that the vector product in equation 16 is very small. We also observe that the magnetic field is symmetric around the center position (position 5), so that the field at position 6 is always similar to the field at position 4, and so on.

For EEG signals, equivalent radial dipoles located at the “crowns” of gyri are generally expected to give the largest signal contributions (Nunez and Srinivasan, 2006). For MEG signals, on the other hand, equivalent current dipoles in brain sulci oriented tangentially to the head surface is expected to provide the largest signals (Hämäläinen et al., 1993). In Figure 5I we thus show the magnetic field with the current dipole moments directed in a tangential direction (that is, in the *y*-direction into the paper in Figure 5F) rather than in the radial direction. In this situation the largest magnetic field component is in the tangential direction $\hat{\theta }$ (around the *y*-axis) in position 5. The $\hat{\varphi }$-component is as expected negligible, while the radial component is antisymmetric around position 5, but negligible in position 5 itself.

Typical magnetic fields measured in human MEG are on the order of 50–500 fT (Hämäläinen et al., 1993), and in Figure 5I we find that magnetic fields of similar magnitudes (~100 fT) are predicted when the current dipole moment from our network is oriented in parallel to the cortical surface. Note, however, that in our model set-up, the dipole is only 11 mm away from the closest MEG sensor at position 5, while in human recordings the minimum distance between tangential dipoles in brain sulci and the MEG sensors may be several centimeters (Hämäläinen et al., 1993). As the magnetic field from a current dipole decays as the square of the distance (see Equation 16), our model likely gives an overestimate of the contribution to the MEG signal from our model network when applied to a human setting.

In Figure 5H we also observe sizable magnetic fields (~20–40 fT) generated by radially-oriented current dipoles. However, the generated fields are in the angular ϕ-direction where the fields have opposite directions on each side of the central position (position 5). Thus, in a setting with several such neighbouring dipoles (generated by neighbouring populations) on cortical gyri, there will be large cancellations effects. Despite the larger distances from the MEG sensors, tangentially oriented dipoles in sulci is therefore expected to dominate the measured MEG in human settings (Hämäläinen et al., 1993).

Animations of EEG surface potentials (color coded) and magnetic field (arrows) of the radially and tangentially oriented current dipole moments are available as Supplementary Video 1 (radial_dipole.mp4) and Supplementary Video 2 (tangential_dipole.mp4), respectively. For the case with a tangential dipole the characteristic “butterfly”-like pattern often seen in MEG recordings is observed (see e.g., Figure 5 in Hämäläinen et al., 1993).

### 3.3. LFPy Parallel Network Performance

In order to assess the performance figures of multicompartment-neuron network implementations in LFPy on a high-performance computing (HPC) facility, we performed a series of simulations with two-population versions of the network presented above. These modified networks consisted only of the layer-5 m-types L5_TTPC1 and L5_MC. We modified cell counts per population *N*_*X*_ and connection probabilities *C*_*YX*_ depending on chosen network population sizes *N*_*X*_ as noted in the text below. All other simulation parameters were kept fixed as given in Tables 1–3.

**Figure 7:**
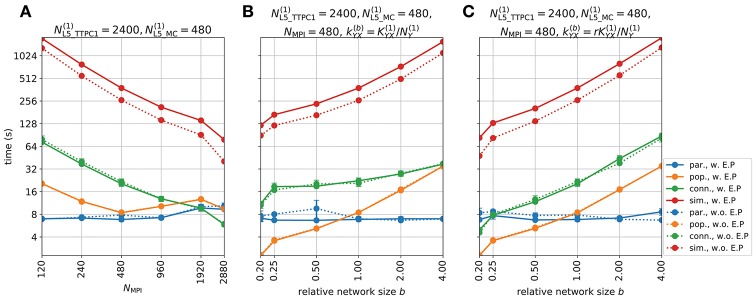
Parallel performance with networks in LFPy. **(A)** Initialization of parameters (par.), population create (pop.), connectivity build (conn.) and main simulation time (sim.) as functions of number of physical CPU cores/MPI processes (*N*_MPI_). The reference network population sizes $N_{X}^{\left(1\right)}$ for *X* ∈ {L5_TTPC1, L5_MC} are given in the panel title. The network was otherwise constructed with synapse, stimulus and connectivity parameters for each possible connection as given in Tables 1–3. Times shown with continuous lines were obtained for simulations that included calculations of extracellular potentials and current dipole moments as in Figures 2–6 (w. E.P.), while times shown with dotted lines were obtained for simulations with no such signal predictions (w.o. E.P.). Each data value is shown as the mean and standard deviation of times obtained from *N* = 3 network realizations instantiated with different random seeds. **(B)** Initialization of parameters, population create, connectivity build and main simulation time as functions of network size relative to the reference network population sizes $N_{X}^{\left(1\right)}$ for *X* ∈ {L5_TTPC1, L5_MC} as given in the panel title. The superset “(1)” denotes a relative network size *b* = 1. Simulations were run using a fixed MPI process count *N*_MPI_ and connection probabilities $C_{YX}^{\left(r\right)}$ were recomputed for different values of *b*, such that the expected total number of connections $K_{YX}^{\left(1\right)}$ was constant between each simulation (using 20). The set-up was otherwise identical to the set-up in **(A)**. **(C)** Same as **(B)**, but with a fixed expected per-cell synapse in-degree $k_{YX}^{\left(r\right)}\equiv rK_{YX}^{\left(1\right)}/N_{Y}^{\left(1\right)}$ across different relative network sizes.

First, we compared set-up times, creation times of populations and connections, and simulation times for instantiations of similarly sized reference networks ($N_{\text{L}5\text{\_}\text{TTPC}1}^{\left(1\right)}=2400,N_{\text{L}5\text{\_}\text{MC}}^{\left(1\right)}=480$) for different number of MPI processes *N*_MPI_ (Figure 7A). *N*_MPI_ was set identical to the number of available physical cores (no multi-threading). A seed value for the random number generator for each network instantiation was varied to obtain an *N* = 3 sample size for each tested value of *N*_MPI_. Both with predictions of extracellular potentials and current dipole moments (continuous lines) and without (dotted lines), the biggest fraction of the total computational time was spent during the main simulation part (red curves), that is, where the simulation is advanced time step by time step. The additional computational cost of computing extracellular potentials and current dipole moments was less than half compared to just simulating the spiking activity in the recurrently connected network. The times spent creating all recurrent connections and synapses (green curves) were between a factor 16 and 32 shorter than the simulation time.

The creation of connections and simulation times scaled strongly with *N*_MPI_. An optimal, or strong, log-log-linear scaling curve can be represented as a function $t\left(N_{\text{MPI}}\right)\propto N_{\text{MPI}}^{-1}$, in particular for *N*_MPI_ ≤ 480, as these *N*_MPI_-values result in an even load balance across parallel processes with the presently used round-robin distribution of cells across MPI processes (see section A2 in Appendix for details). Each parallel process has the same number of cells of each m-type, segments ($n_{j}^{\text{seg}}$) and state variables corresponding to different active ion-channel models. Only variations in per-cell in-degrees (synapse counts) across different processes and simulations occurred due to the random network connectivity model, but even with different random seeds in each trial the trial variability was small (error bars denoting standard deviations are hardly seen).

The creation of populations (orange curves) however showed worse scaling behaviour for *N*_MPI_ > 480, in part due to uneven load balance. Another possible reason for reduced performance was the increased strain on the file system as all processes simultaneously access the same single-neuron source files upon instantiating individual NetworkCell objects. This might have been avoided by creating local copies of the necessary files on each compute node, but we did not pursue this here as the overall time spent instantiating neuron populations was only a fraction of the observed simulation times. The loading of parameters and other needed data (blue curves) was, as expected, fairly constant for different values of *N*_MPI_ as we did not parallelize the corresponding code.

As a second scaling-performance test, we ran series of simulations with *N*_MPI_ = 480 but varied the total network size by a factor *b* ∈ {0.2, 0.25, 0.5, 1, 2, 4} while keeping the expected number of connections *K*_*YX*_ (and thus the number of synapses) between pre- and post-synaptic populations *X* and *Y* fixed (Figure 7B). The expected number of randomly created (binomially distributed) connections *K*_*YX*_ was calculated using the relation (Potjans and Diesmann, 2014):

(20) $$\begin{array}{l} C_{YX}=1-{\left(1-\frac{1}{N_{X}N_{Y}}\right)}^{K_{YX}}\;, \end{array}$$

with reference network size ($N_{\text{L}5\text{\_}\text{TTPC}1}^{\left(1\right)}=2400,N_{\text{L}5\text{\_}\text{MC}}^{\left(1\right)}=480$) and connection probabilities *C*_*YX*_ as given in Table 3. Similar to the test presented in Figure 5A, most of the total computation time was spent during the main simulation part (red curves), followed by creation of connections (green curves) and loading of different parameters (blue curves).

In contrast to the previous case, the creation of cells in the network displayed strong scaling with network size (which implies a relationship *t*(*r*)∝*b*). The supra-optimal scaling seen for connections can be explained by the creation of similar connection counts across different factors *b*. (Note that supra-optimal scaling implies that *t*(*r*)∝*b*^*q*^ with exponent *q* ∈ (0, 1), while sub-optimal scaling implies that *q* > 1.) For the tested factors *b* = 0.25 and *b* = 0.5 we expected sub-optimal scaling for creating populations and connections, as well as for simulation duration. These *b*-values gave different cell counts and thus inhomogeneous load-balances across MPI processes, which was unavoidable with the presently used round-robin parallelization scheme. A jump in performance was seen for *b* = 0.2 which resulted in only one multicompartment neuron and corresponding calculations on each MPI process.

As a third scaling-performance test we fixed the mean per-cell synapse in-degree *k*_*YX*_ (count of incoming connections per cell) and reran network simulations for different network sizes (Figure 7C). The total number of connections was thus set to $bK_{YX}^{\left(1\right)}$ and corresponding connection probabilities *C*_*YX*_ were recomputed accordingly using equation 20. As expected, this modification mostly affected the time spent creating connections (green curve), and resulted in a near-linear performance curve for scaling factors *b* ≥ 1.

**Figure 8:**
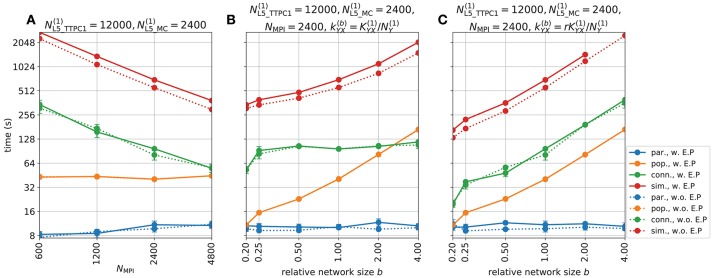
Parallel performance with networks in LFPy II. **(A)** Similar to Figure 7A, but with network population sizes upscaled by a factor 5, and a corresponding increase in parallel job sizes. **(B,C)** Similar to Figures 7B,C, but with network population sizes and parallel job sizes increased by a factor 5.

As a final performance assessment we repeated the experiment described above with upscaled networks and increased MPI pool sizes. In Figure 8A we set the reference network population sizes $N_{\text{L}5\text{\_}\text{TTPC}1}^{\left(1\right)}=12,000$ and $N_{\text{L}5\text{\_}\text{MC}}^{\left(1\right)}=2,400$ and varied *N*_MPI_ between 600 and 4,800. LFPy's parallel performance was strong also here, and Figure 8A consequently shows trends similar to the findings for the smaller network. Here, the time spent creating populations (orange curves) was reasonably invariant for different *N*_MPI_ values, and increased overall by some factor 2–4 compared to the previous case. The parameter loading times were similar, while the time spent connecting the network was increased by a factor ~ 4, but the simulation times increased only by a factor ≲ 2. The differences in connection and simulation times seen here, can be explained by the fact that the typical synapse in-degrees were not preserved. Instead, the synapse in-degrees were increased for the larger network, as we used the connection probability values defined in Table 3.

In Figures 8B,C we set *N*_MPI_ = 2, 400, and varied the network population sizes relative to the reference network population sizes in Figure 8A by the factor *b* ∈ {0.2, 0.25, 0.5, 1, 2, 4}. Again, the performance figures were in qualitative agreement with the previous results for the smaller network and smaller MPI pool sizes. The population creation times and simulation times with and without signal predictions displayed strong scaling with relative network size. The time spent loading parameters was increased by a small amount (by a factor ≲ 2), which likely reflected the increased strain on the file and communication system on the cluster, due to larger MPI pool sizes. The times spent creating the populations were also here near ideally dependent on *N*_MPI_ in both Figures 8B,C. As the total number of connections (and synapses) were conserved across network population sizes in Figure 8B, the connection times varied only by a factor two from the smallest to the largest network. In Figure 8C, where the number of connections per neuron was kept approximately constant, a doubling in network size resulted in a doubling in connection times. The larger network simulations required approximately twice the amount of time, compared to the smaller network simulations in Figure 7. In Figure 8C, simulations with LFP predictions consistently failed for the largest network size (*b* = 4), most likely due to lack of available memory to create arrays for storing current dipole moments and extracellular potentials with the increased count of instantiated connections.

## 4. Discussion

In the present paper we have presented LFPy 2.0, a majorly revised version of the LFPy Python package with several added features compared to its initial release (Lindén et al., 2014).

### 4.1. New Features in LFPy 2.0

The first version of LFPy only allowed for the computation of electrical measurements from activity in single neurons or, by trivial parallellization, populations of neurons only receiving feedforward synaptic input. LFPy 2.0 allows for simulations of recurrently connected neurons as well, for example the types of neuronal networks in cortex. Further, the first version of LFPy was tailored to compute extracellular potentials (spikes, LFPs) inside the brain. Here it was assumed that all active neurons were embedded in an infinite *homogeneous* (i.e., same extracellular conductivity everywhere) and *isotropic* (i.e., same extracellular conductivity in all directions) volume conductor (section 2.2.1). LFPy 2.0 includes several new features and measures of neural activity:

- Stepwise discontinuities in the extracellular conductivity, such as at the cortical surface, can be included by means of the Method-of-Images (section 2.2.2) to compute potentials immediately below or on the cortical surface (i.e., electrocorticographic recordings; ECoG). This approach can also be applied in the computation of potentials recorded by microelectrode arrays (MEAs) (Ness et al., 2015).
- Cylindrical anisotropic conductivity (section 2.2.3) can be included in the computation of spikes and LFPs, reflecting for example that in cortex and hippocampus the conductivity might be larger in the depth direction (along the apical pyramidal-neuron dendrites) than in the lateral directions (Goto et al., 2010).
- Current dipole moments from single neurons and populations of neurons are computed (section 2.3.1) for later use in calculation of signals of systems-level electrical and magnetic recordings (EEG, ECoG, MEG), also for more detailed head models than what is considered presently in LFPy 2.0 (as described in next two items).
- Electrical potentials at the scalp (electroencephalographic recordings; EEG) are computed from the current dipole moments and spherical head models, in particular the four-sphere head model (Nunez and Srinivasan, 2006; Næss et al., 2017), cf. section 2.3.3. This four-sphere head also predicts ECoG signals (section 2.3.4).
- Magnetic fields outside the head (magnetoencephalographic recordings; MEG) can be computed from the current dipole moments assuming a spherically symmetric head model (section 2.3.5). Likewise, magnetic field inside the brain can be computed directly from neuronal axial currents (section 2.4).

LFPy 2.0 also includes much more rigorous code testing with more than 270 unit tests, automated build testing with TravisCI (travis-ci.org/LFPy/LFPy) with different versions of Python (2.7, 3.4-3.6), test coverage of code using coveralls (coveralls.io/github/LFPy/LFPy), automated documentation builds using Read the Docs (lfpy.readthedocs.io), and several updated example files, as well as new examples demonstrating different scientific cases using the new functionalities. The software runs on a wide variety of operating systems, including Linux, Mac OS and Windows.

### 4.2. Example Applications

To illustrate some of the new measurement modalities incorporated in LFPy 2.0 we showed in Figure 2 the LFP and EEG signature of a simple pyramidal-like neuron receiving a single excitatory synaptic input on its apical dendrite. In this example the extracellular medium was assumed to be homogeneous, and a characteristic dipolar profile was observed in the extracellular potential (Figure 2B). The accuracy of the far-field electrical dipole approximation (Equation 15) for distances of a few millimeters or more away from the neuronal source, was also demonstrated. The corresponding magnetic field set up by the neuron (Figure 2C) was quite distinct from the electric potential pattern, but also here far-field magnetic dipole approximation (Equation 16) was observed to be accurate some distance away.

To illustrate the implementation of networks in LFPy 2.0 we show in section A2 (Appendix) a code example for a small network using simplified ball-and-stick neurons connected by conductance-based synapses. Our main example applications were on a network of about 5,500 morphologically and biophysically detailed neuron models from the reconstructed somatosensory cortex column of Markram et al. (2015), connected using probabilistic synapse models with short-term plasticity. For this example, Figure 5 provided results for a one-second epoch of network activity where spikes (Figures 5A,B), LFPs inside the cortical model column (Figure 5C), the ECoG signal recorded at cortical surface ( Figure 5D), and the net current dipole moment (Figure 5E) were depicted. The computed current dipole moment was further used to compute the corresponding EEG signal with the four-sphere head model for the situation where the model network was placed on top of a cortical gyrus where the apical dendrites of the pyramidal neurons, and thus the current dipole moment, is pointing in the radial direction (Figure 5G). The same current dipole moment was also used to compute the MEG signal, assuming a spherically-symmetric head volume-conductor model, both for the case when the net current dipole is directed perpendicular (Figure 5H) and parallel (Figure 5I) to the scalp. The latter situation could correspond to the case where the model network is positioned in a cortical sulcus.

While the example network was set up mainly to demonstrate the new features in LFPy 2.0, some of the example results are notable. As expected the two excitatory pyramidal cell populations in the network provided almost all of the recorded LFP signal (except in the deep layers where the layer-5 inhibitory Martinotti-cell population also gave a sizable contribution), cf. Figure 6E. Likewise, the two excitatory pyramidal cell populations also gave the dominant contributions to the net current dipole moment providing the EEG and MEG signals (Figure 6J).

For the present example network comprising about 5000 pyramidal neurons, we observed the maximum magnitude of the EEG signal to be about 0.1 μV (Figure 5G), that is, about two orders of magnitude smaller than the typical size of measured EEG signals of ~10 μV (Nunez and Srinivasan, 2006, Figure 1.1). Thus our example model network appears too small, that is, it incorporates too few pyramidal neurons, to account for the typical experimentally recorded EEG signal amplitudes.

The maximum magnetic field computed at the cortical surface was seen in Figures 5H,I to be about 100 fT, that is, similar in magnitude to typical magnetic fields measured by MEG sensors in a human setting (~50–500 fT, Hämäläinen et al., 1993). However, our model predictions assumed the minimum distance between the current dipoles and the magnetic-field recording device to be only about a centimeter, likely much smaller than the typical minimal distance between the dominant tangential dipoles in cortical sulci and the human MEG sensors. Since the magnetic field around a current dipole decays as the square of the distance, our modeling likely substantially overestimates the magnetic field that would produced by the computed current dipoles in a human setting.

### 4.3. Use of LFPy

#### 4.3.1. Comparison of Candidate Models With Experiments

An obvious application of LFPy is, following the tradition of physics, to (i) compute predictions of the various available measures of neural activity from different candidate models and (ii) identify which model, or which class of models, is in best agreement with the experimental data. While not always possible, the approach is preferably pursued on multimodal data measured simultaneously (for example simultaneous recordings of spikes, LFP and ECoG). The multi-objective comparison of experimental data with candidate models is a subject on its own, and will not be discussed here (but see, for example, Druckmann et al., 2007).

#### 4.3.2. Validation of Data Analysis Methods

Neuroscience relies on data analysis, and data analysis methods should be validated (Denker et al., 2012). An important application of LFPy could be to provide model-based ground-truth benchmarking data for such validation. This approach has already been used with biophysically detailed neuron models to test methods for spike sorting (Einevoll et al., 2012; Hagen et al., 2015; Lee et al., 2017), neuron classification (Buccino et al., 2017), estimation of firing rates from multi-unit activity (MUA) (Pettersen et al., 2008), current-source density (CSD) analysis (Pettersen et al., 2008; Łęski et al., 2011; Ness et al., 2015), independent component analysis (ICA) (Głąbska et al., 2014) and laminar population analysis (LPA) (Głąbska et al., 2016). Other analysis methods to consider are for example EEG and MEG source localization methods, for example as provided by open-source projects like MNE (martinos.org/mne, Gramfort et al., 2013, 2014), BrainStorm (neuroimage.usc.edu/brainstorm, Tadel et al., 2011), EEGLAB (sccn.ucsd.edu/eeglab, Delorme and Makeig, 2004), Fieldtrip (fieldtriptoolbox.org, Oostenveld et al., 2011), nutmeg (nutmeg.berkeley.edu/, Dalal et al., 2004) and SPM (fil.ion.ucl.ac.uk/spm) where LFPy 2.0 can be used to generate benchmarking data with known “ground truth.”

Likewise, LFPy could be used to aid in the interpretation of various statistical measures of electrophysiological activity such as *spike-triggered LFP* or *mutual information* (Einevoll et al., 2013). The interpretation of these measures in terms of the underlying neural network activity is a priori not trivial, but intuition and understanding can be gained by LFPy model investigations where simulation results can be compared with neural activity directly. An example of this was given in Hagen et al. (2016). There the spike-triggered LFP as measured in the model simulation was compared with other ways of accounting for spike-LFP relationships with a simpler physical explanation, that is, the LFP signature following activation of a presynaptic neural population.

It should be noted that the LFPy network model does not necessarily have to be finely tuned to a particular experimental system in order for it to be suitable for validation of data analysis methods: Methods claimed to have fairly general applicability should also be applicable to biologically plausible example network models.

#### 4.3.3. Testing of Simplified Modeling Schemes

LFPy now allows for the concurrent simulation of intracellular (membrane potential) and extracellular signals (spikes, MUA, LFP, EEG, MEG) for recurrent networks of biophysically and morphologically detailed neuron models. Such network models are computationally demanding to run (Markram et al., 2015), in particular when extracellular signals are computed simultaneously (Reimann et al., 2013). A computationally less demanding alternative is a *hybrid LFP* scheme where the network dynamics, that is, spikes, are modeled with simple point-neuron models such as the integrate-and fire model, and the stored spikes are played back in a second computational step computing the extracellular potentials using multicompartment neuron models (Mazzoni et al., 2015; Hagen et al., 2016).

This scheme requires that salient features of spiking activity of networks of detailed multicompartment neuron models can be accurately captured by point-neuron network models. This was for example demonstrated by Rössert et al. (2016) who reproduced key network behaviour of a reconstructed somatosensory column (Markram et al., 2015) by systematic mapping of synaptic input to somatic responses in generalized leaky integrate-and-fire neurons. Likewise, the accuracy of the second step in the hybrid scheme where the extracellular potential is computed, can be systematically tested by comparing resulting predicted extracellular potential with the ground-truth potentials provided by LFPy. The same approach can also be applied to test other simplified schemes for computing extracellular signals.

### 4.4. Possible Refinements of Measurement Models in LFPy

#### 4.4.1. Frequency-Dependence of Extracellular Conductivity

The present forward-modeling schemes for electrical potentials assume the extracellular conductivities σ_e_ to be independent of frequency. If such a frequency dependence is found and described, it can in principle be straightforwardly incorporated by considering each frequency (Fourier) component of recorded signal independently. This was, for example, pursued in Miceli et al. (2017) where each frequency component of the spikes and LFP signals were computed independently (i.e., each frequency component had a specific value of σ_e_ and a corresponding phase shift required by the Kramers-Kronig relations to preserve causality) and eventually summed to provide the full electric potential. However, on balance the experimental evidence points to at most a weak frequency dependence of σ_e_ with only minor putative effects on the recorded spikes and LFPs (Miceli et al., 2017). Therefore, the present approximation in LFPy 2.0 to assume a frequency-independent conductivity σ_e_, seems warranted.

#### 4.4.2. Modeling of ECoG Signals

LFPy 2.0 provides two different methods for computing ECoG signals, that is, signals at the cortical surface: the method-of-images (MoI) section 2.2.2 and the four-sphere model section 2.3.4 which both have their pros and cons. The MoI method assumes a planar cortical interface and that the media above this interface can be described electrically by means of a single isotropic electrical conductivity. The four-sphere model assumes a spherical cortical surface and uses the far-field dipole approximation which requires the dipolar sources to be sufficiently far away from the recording contacts. With the present use of current dipole moments representing entire neuron populations, this approximation is challenged by the relatively short distance between in particular the most superficial populations and the cortical surface (Næss, 2015). A future project is to systematically explore the accuracy of these two methods for ECoG modeling, for example by comparing their predictions for different situations.

The present forward modeling of electrical potentials are based on stylized spatial (planar/spherical geometries, step-wise varying conductivities) and directional (isotropy/cylindrical anisotropy) variations. More complicated models for the variation of the extracellular conductivity can be accounted for by means of finite-element modeling (FEM, Logg et al., 2012; Lempka and McIntyre, 2013; Ness et al., 2015; Næss et al., 2017) for which the “lead field,” that is, the contribution from transmembrane currents or dipole moments to electric signals, always can be computed (Malmivuo and Plonsey, 1995). FEM could, for example, be used to explore in detail how the recording device affects the recorded ECoG signal when a grid of ECoG contacts are embedded in an insulating material (see for example, Castagnola et al. (2014)), in analogy to the study of multielectrode arrays (MEAs) in Ness et al. (2015).

#### 4.4.3. More Complicated Head Models

The current dipole moments computed by LFPy can also be used to compute EEG and MEG signals based on geometrically detailed head models measured by MRI (Bangera et al., 2010; DeMunck et al., 2012; Vorwerk et al., 2014; Huang et al., 2016). Note, however, that geometrically detailed head models do not automatically transfer to electrically detailed head models, and it is thus not always clear how much accuracy is gained by using such models rather than the simpler head models currently implemented in LFPy (see discussion in Nunez and Srinivasan, 2006, Ch. 6).

### 4.5. Possible Improvements of LFPy Code

While we here demonstrated a relatively strong scaling of parallel network implementations in LFPy, the code itself could be further optimized for improving overall simulation speeds and reduced memory consumption allowing for larger networks for any given MPI pool size.

One common way of improving efficiency of Python applications is rewriting “slow” code to use Cython (C-extensions for Python, cython.org, Smith, 2015). The current LFPy version uses Cython to a limited extent, but remaining code bottlenecks could be identified and addressed accordingly. One potential problem with efficient porting of parts of LFPy's Python code to Cython is repeated calls to NEURON's Python interface, which from a performance point of view should be avoided.

One known bottleneck with parallel implementations of multicompartment neuron networks is uneven load balance, resulting from the fact that individual neurons with very uneven numbers of compartments may be assigned to the different MPI processes. Uneven load balance could potentially be addressed by incorporating the multi-split method described in Hines et al. (2008), as it appears compatible with the presently used CVode.use_fast_imem() method (available since NEURON v7.4). LFPy could then be updated accordingly.

Even without the NEURON multi-split method, distribution of cells among MPI processes using a round-robin scheme could, however, be optimized to level out large differences in compartment counts (and corresponding numbers of state variables). Memory consumption could also be addressed by choosing more efficient memory structures or generators, for example, for connectivity management, and by avoiding in-memory storage of output data wherever possible. File-based I/O operations during ongoing simulations may, however, come at the expense of increased simulation times.

In terms of improved support for simulator-independent (agnostic) model description languages for neuronal models such as NeuroML (Gleeson et al., 2010; Cannon et al., 2014) or NESTML (Plotnikov et al., 2016), LFPy's TemplateCell and NetworkCell classes already now support loading of active and passive single-neuron model files translated to NEURON's HOC and NMODL languages from NeuroML and NeuroML2 (now in development). A growing number of such single-neuron models is becoming available through, for example the Open Source Brain initiative (opensourcebrain.org), which can readily be used in order to construct new network models. While certainly doable, LFPy is at present not set up for automatic loading of entire neuron networks specified in NeuroML. Also, single-cell and network models specified using LFPy could, in principle, be possible to translate into NeuroML as well, which would allow for executing such models using for example NetPyne (netpyne.org) or LEMS (Cannon et al., 2014).

### 4.6. Other Measurement Modalities in LFPy

The present version of LFPy only models recording of electric and magnetic brain signals. Optical recording methods are now frequently used in neurophysiology, however, and forward-modeling of such signals would be a natural extension of the present functionality. In voltage-sensitive dye imaging (VSDi), the recorded signals reflects a weighted average of the membrane potentials, and such averages can be readily computed since the membrane voltages in all neuronal compartments are computed during a network simulation simulation (Chemla and Chavane, 2010a,b). This must then be combined with proper forward-modeling of the propagation of the light through the brain tissue (Tian et al., 2011; Abdellah et al., 2015, 2017).

Calcium imaging has become a wide-spread method for measuring neural dynamics (Grienberger and Konnerth, 2012). With the use of neuron models that explicitly includes dynamic modelling of the intracellular calcium concentrations (for example, Hay et al., 2011; Almog and Korngreen, 2014) such signals could be directly modeled as well.

### 4.7. Alternatives to LFPy

For the purpose of computing extracellular potentials under the assumption of homogeneous extracellular conductivity and networks of multicompartment neuron models, some alternatives to LFPy 2.0 exist. Genesis (genesis-sim.org, Bower and Beeman, 1998) incorporates the simple point-source formalism (Equation 4), while the MATLAB tool Vertex (vertexsimulator.org, Tomsett et al., 2015) allows for computing extracellular potentials but not for multicompartment neuron models with arbitrary levels of detail. The MOOSE simulator (https://moose.ncbs.res.in, Ray and Bhalla, 2008) do not appear to natively incorporate electrostatic forward models. An extension to NEURON named LFPsim (github.com/compneuro/LFPsim, Parasuram et al., 2016) supports single neurons and networks but relies on the NEURON GUI. This may allow for simple evaluation of LFPs generated by small networks, but hampers application to large-scale networks running in parallel.

The Python and NEURON based tools NetPyne (netpyne.org) and BioNet (github.com/AllenInstitute/bmtk, Gratiy et al. (2018)), part of the Allen Brain Institute's Brain Modeling Toolkit, do, however, support biophysically detailed networks of neurons running in parallel with predictions of extracellular potentials, but presently without the wider range of electric and magnetic forward models now provided in LFPy 2.0. Similar to LFPy 2.0, high-level functionality to specify networks are provided to simplify the generation of networks.

Finally, the recent ‘Human Neocortical Neurosolver’ (hnn.brown.edu) can compute LFP, MEG and EEG signals, but has a focus on signals generated by specific generic cortical network topologies, namely using neurons with few compartments organized in two “cortical layers” 2/3 and 5. In contrast, LFPy 2.0 supports defining networks with an arbitrary number of layers and biophysical detail.

### 4.8. Outlook

While information in the brain might largely be represented by spike trains, we believe that tools such as LFPy will be instrumental in testing candidate network models aiming to account for this information processing. In the foreseeable future, experimental data against which candidate models can be tested will be a limiting factor. It is thus key that such candidate models can be tested not only against spike trains, but also other measurement modalities.

This updated version of LFPy makes a major step toward being a true multi-scale simulator of neural circuits, allowing for flexible incorporation of highly detailed neuron models at the micrometer scale, yet able to also predict recorded signals such as EEG and MEG at the systems-level scale. The largest network considered here had 57,600 neurons. With the present code, not optimized for numerical efficiency, the simulation of 1.5 s of biological time on this network required about 1,600 CPU hours across 2,400 MPI processes. With optimized code, we expect that much larger networks can soon be addressed routinely as ever more powerful computers gradually become available. The software is also publicly available on GitHub and retains the open-source software license of its initial release, and our hope is that continued development remains driven by needs and contributions of individuals and groups of researchers.

## Data Availability Statement

All source codes to reproduce the simulation results of this publication are available as example files in the main LFPy GitHub repository (github.com/LFPy/LFPy). The stable release of LFPy 2.0.0 is deposited on zenodo.org (doi.org/10.5281/zenodo.1401088).

## Author Contributions

EH and SN wrote the first paper draft. EH, SN, TN, and GE co-wrote the paper. EH implemented and ran all simulations. SN implemented the four-sphere head model for the EEG and axial current calculations. TN implemented the LFP models for anisotropic and inhomogeneous (Method-of-Images) electrical conductivity.

### Conflict of Interest Statement

The authors declare that the research was conducted in the absence of any commercial or financial relationships that could be construed as a potential conflict of interest.

## Appendix

### A1. Algorithms

**Algorithm 1:** Axial current calculations in LFPy 2.0

1:	children = dict. of child indices *n* of each parent section sec
2:	connections = dict. of relative location χ ∈ [0, 1] where children connect onto parent section
3:	**R**^i^ = list of axial resistances of child segments to corresponding parent nodes
4:	**V**^m^ = list of membrane potentials at midpoints of each segment
5:	Initialize length 2(*n*^seg^ − 1) lists **I**^a^, **d** and **r** indexed by *m* ∈ {0, 1, …, 2(*n*^seg^ − 1) − 1}
6:	set current and segment indices *m* = *n* = 0
7:	set root_sec = True
8:	**for** sec in neuron morphology **do**
9:	**if** sec has parent section **then**
10:	set parent segment index *f* from children and connections
11:	set init_seg = True and root_sec = False
12:	**if** count(children[sec])>1 **then**
13:	set branch = True
14:	set χ = connections[sec]
15:	**for** seg in sec **do**
16:	**if** root_sec **then**
17:	set *n* = 1 and *f* = 0
18:	set init_seg = False and root_sec = False
19:	**continue**
20:	set $R_{fn}^{\text{i}}={\text{R}}^{\text{i}}\left[n\right]$
21:	set $V_{f}^{\text{m}}={\text{V}}^{\text{m}}\left[f\right]$
22:	set $V_{n}^{\text{m}}={\text{V}}^{\text{m}}\left[n\right]$
23:	**if not** init_seg **or** 0 < χ < 1 **then**
24:	compute ${\text{I}}^{\text{a}}\left[m\right]=\left(V_{f}^{\text{m}}-V_{n}^{\text{m}}\right)/R_{fn}^{\text{i}}$ (see **Figure 3B,C,E**)
25:	**else**
26:	set $R_{n}^{\text{i}}={\text{R}}^{\text{i}}\left[n\right]$ (axial resistance from mid to start point of segment *n*)
27:	set $R_{f}^{\text{i}}$ (axial resistance from end to mid point of parent segment *f*)
28:	**if not** branch **then**
29:	compute ${\text{I}}^{\text{a}}\left[m\right]=\left(V_{f}^{\text{m}}-V_{n}^{\text{m}}\right)/\left(R_{f}^{\text{i}}+R_{n}^{\text{i}}\right)$ (see **Figure 3D**)
30:	**else**
31:	compute branch point potential
	$$\begin{array}{l} V_{\times }^{\text{m}}=\frac{\sum_{h}V_{h}^{\text{m}}/R_{h}^{\text{i}}}{\sum_{h}1/R_{h}^{\text{i}}}\text{ for }h\in \left\{f,n_{1},n_{2},\ldots n_{\text{children}}\right\} \end{array}$$
32:	compute ${\text{I}}^{\text{a}}\left[m\right]=\left(V_{\times }^{\text{m}}-V_{n}^{\text{m}}\right)/R_{n}^{\text{i}}$ (see **Figure 3F**)
33:	set **I**^a^[*m*+1] = **I**^a^[*m*]
34:	compute **d**[*m*] by subtracting the midpoint of *f* from the start point of *n*
35:	compute **d**[*m*+1] by subtracting the start point of *n* from the midpoint of *n*
36:	compute **r**[*m*] by subtracting 0.5·**d**[*m*] from the start point of *n*
37:	compute **r**[*m*+1] by subtracting 0.5·**d**[*m*+1] from the midpoint of *n*
38:	set *f* = *n, n* = *n*+1 and *m* = *m*+2
39:	set branch = False and init_seg = False

### A2. New Classes and Network Use-Case Implementation

The first release of LFPy described in Lindén et al. (2014) included a set of Python class definitions for instantiating single-cell models (Cell, TemplateCell) and corresponding instrumentation of the models with synapse point processes attached to the cell (Synapse), patch-clamp electrodes (StimIntElectrode) and extracellular recording electrodes (RecExtElectrode). Simulations with multiple simultaneous cell-object instances were at the time not supported. Class TemplateCell supported the use of template specifications, a requirement for networks in NEURON, but was primarily written to support source codes of ‘network-ready’ single-cell models such as the Hay et al. (2011) models of layer-5 pyramidal neurons available from, for example, ModelDB (senselab.med.yale.edu/modeldb, McDougal et al. (2017)).

The “one cell at a time” approach may seem limited, in particular when considering ongoing network interactions, but knowing that forward-modeling of extracellular potentials can be decoupled from the network simulation, users could always set up simulations of each individual cell, play back synapse activation times as occurring in the connected network, and sum up the single-cell contributions to the extracellular potential. Thus, the calculation of extracellular potentials can even be dealt with in an “embarrassingly” parallel manner (Foster, 2007; Hagen et al., 2016). These simplifying steps are not possible if the extracellular potential itself affects the cellular dynamics, that is, if mutual interactions between cellular compartments belonging to the same or different cells occur through the extracellular potential, so-called ephaptic interactions (Anastassiou et al., 2011; Goldwyn and Rinzel, 2016; Tveito et al., 2017).

For the present LFPy 2.0 release, we added support for simulations of recurrently connected multicompartment models with concurrent calculations of extracellular potentials and current dipole moments. As described above, the current dipole moment is used for predictions of distal electric potentials (for example scalp surface potentials as in EEG measurements) and magnetic fields (as in MEG measurements). For our example use case, we considered a recurrent network of four populations of multicompartment neuron models. We added a new set of generic class definitions in LFPy to represent the network, its populations and neurons, as well as classes representing different volume-conductor models and measurement modalities as summarized next.

*Cells:* Each individual neuron in an LFPy network exists as an instantiation of class NetworkCell. As this class definition uses class inheritance from the old TemplateCell and in turn Cell classes, it retains all common methods and attributes from its parent classes. The NetworkCell can therefore be instantiated in a similar manner as its parent class (plotted output not shown):

The morphology and template files referred to above are defined in NEURON “hoc” language files. A “ball and stick” style morphology file with active soma (Hodgkin & Huxley Na^+^, K^+^ and leak channels) and passive dendrite sections and corresponding template file was written as:

and

In contrast to class TemplateCell, class NetworkCell has built-in methods to detect somatic action potentials and set-ups of synapses being activated by such threshold crossings in other cells.

*Network populations:* One step up in the hierarchy, class NetworkPopulation represents a size *N*_*X*_ population of NetworkCell instantiations of one particular cell type (*X*) in the network. The class can be used directly as (print output not shown):

Direct instantiation of class NetworkPopulation, however, is of limited use as it does not provide any means of simulation control by itself, and has only one built-in method to draw and set random cell-body positions within a chosen radius (pop_args[‘radius’]) and depth from the normal distribution $N\left(\bar{u},\sigma_{\bar{u}}\right)$. In the code example above, pop_args[‘loc’] refers to expected mean depth $\bar{u}$ and pop_args[‘scale’] to the corresponding standard deviation $\sigma_{\bar{u}}$. A random cell rotation around its own vertical *z*-axis is applied by default. The integer cell.gid value accessed above is a unique global identifier *g*_id_ of each cell in the network, and is assigned in running order from the number first_gid. For parallel execution using MPI, cells will be distributed among threads according to the round-robin rule if the condition *g*_id_*%N*_MPI_ = = *k* is True, where % denotes a division modulus operation, *N*_MPI_ the MPI pool size and *k* ∈ [0, 1, …, *N*_MPI_ − 1] the corresponding rank number.

*Networks:* The new network functionality is provided through class Network. An instantiation of the class sets attributes for the default destination of file output, temporal duration *t* and resolution *dt* of the simulation, a chosen initial voltage $V_{\text{init}}^{\text{m}}$ and global temperature control *T*_celsius_ (which affects channel dynamics). Furthermore, the class instance provides built-in methods to create any number of *N*_*X*_-sized populations *X*. Different built-in class methods create random connectivity matrices ${\text{C}}_{XY}^{\left(r\right)}$ (per rank, see *Connectivity Model* in section Section 2.5) between any presynaptic population *X* and postsynaptic population *Y*, and connect *X* and *Y* using an integer number of synapses per connection *n*^syn^ drawn from the capped normal distribution $N\left({\bar{n}}^{\text{syn}},\sigma_{\bar{n}}^{\text{syn}}\right)H\left(n\right)$ where *H*(·) denotes the Heaviside step function. Similarly, synaptic conductances *g*^syn^ are drawn from the distribution $N\left({\bar{g}}^{\text{syn}},\sigma_{\bar{g}}^{\text{syn}}\right)H\left(g-g_{\text{min}}\right)$ (where *g*_min_ denotes minimum synaptic conductance) with connection delays δ^syn^ from $N\left({\bar{\delta }}^{\text{syn}},\sigma_{\bar{\delta }}^{\text{syn}}\right)H\left(\delta -\delta_{\text{min}}\right)$ (where δ_min_ denotes the minimum delay in the network). The network class handles the synapse model in NEURON and corresponding parameters (time constants, reversal potentials, putative synapse locations etc.), and finally provides a simulation control procedure. The simulation control allows for concurrent calculation of network activity and prediction of extracellular potentials as well as the current dipole moment.

In order to set up a complete network simulation we may choose to define NetworkCell and NetworkPopulation parameters as above, and define parameter dictionaries for our instances of Network and extracellular measurement device RecExtElectrode:

Furthermore, we define population names (*X*) and corresponding sizes (*N*_*X*_), as well as one overall connection probability (*C*_*YX*_):

Then, we may chose to define the synapse model and corresponding parameters (here using NEURON's built-in two-exponential model Exp2Syn) for synapse conductances (weight), delays and synapses per connection (multapses), as well as layer-specificities of connections ($L_{YXL}$, see Hagen et al., 2016 and below):

Note that we above relied on Python list-comprehension tricks for compactness. Having defined all parameters, one can then create the network, populations, stimulus, connections, recording devices, run the simulation and collect simulation output:

The argument SPIKES returned by the final network.simulate method call is a dictionary with keys gids and times, where the corresponding values are lists of global neuron ID's (*g*_ID_) and numpy arrays with spike times of each respective unit in the network. The returned OUTPUT and DIPOLEMOMENT arguments are numpy arrays with structured datatypes (sometimes referred to as record arrays). The array OUTPUT[‘imem’] is the total extracellular potential from all transmembrane currents in units of mV, the entries ‘E’ and ‘I’ contributions from the excitatory and inhibitory neuron populations, respectively. DIPOLEMOMENT similarly contains the current dipole moment from populations ‘E’ and ‘I’, but not the sum as the current dipole moment of different populations may, in principle, be freely positioned in different locations within a volume conductor. The computed current dipole moments by themselves have no well defined positions in space and must explicitly be assigned a position by the user, unlike the individual compartment positions used when computing the extracellular potential.

The corresponding LFPy 2.0 example files discussed throughout this section are:

- /examples/example_network/example_network_cell.py,
- /examples/example_network/example_network_population.py
- /examples/example_network/example_network.py.
